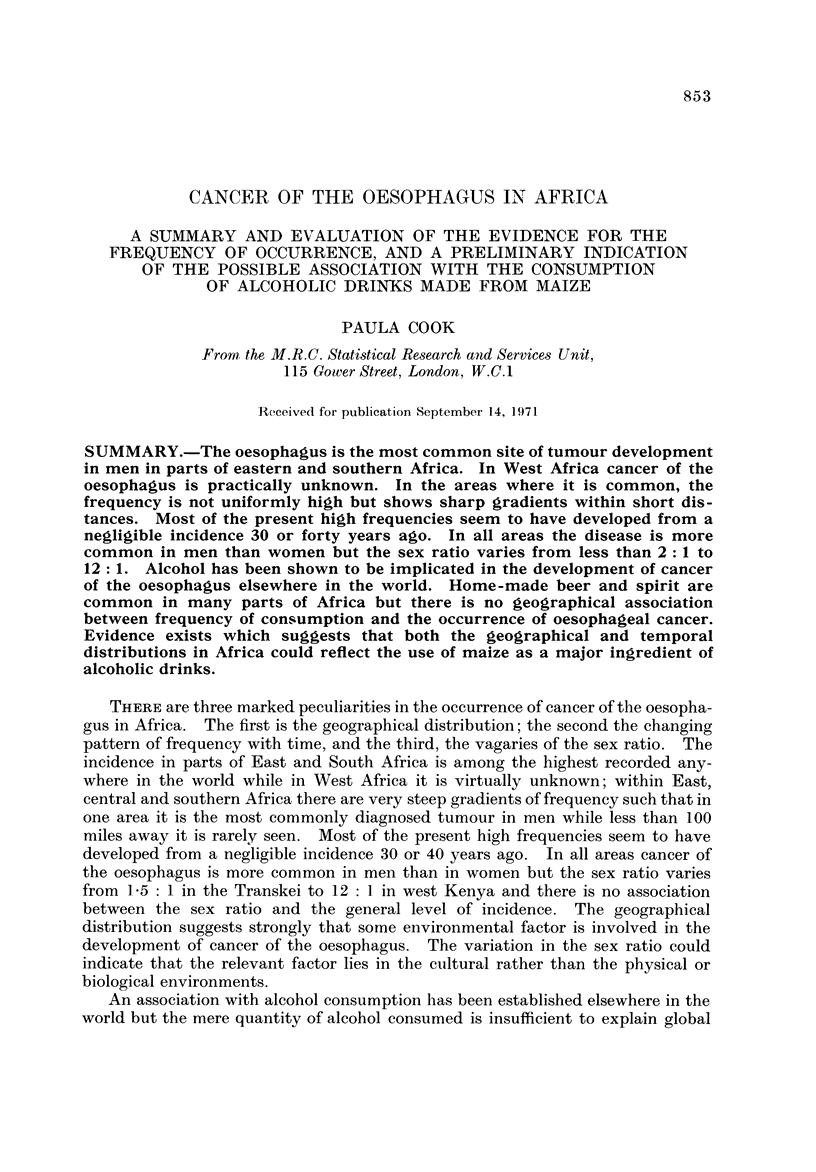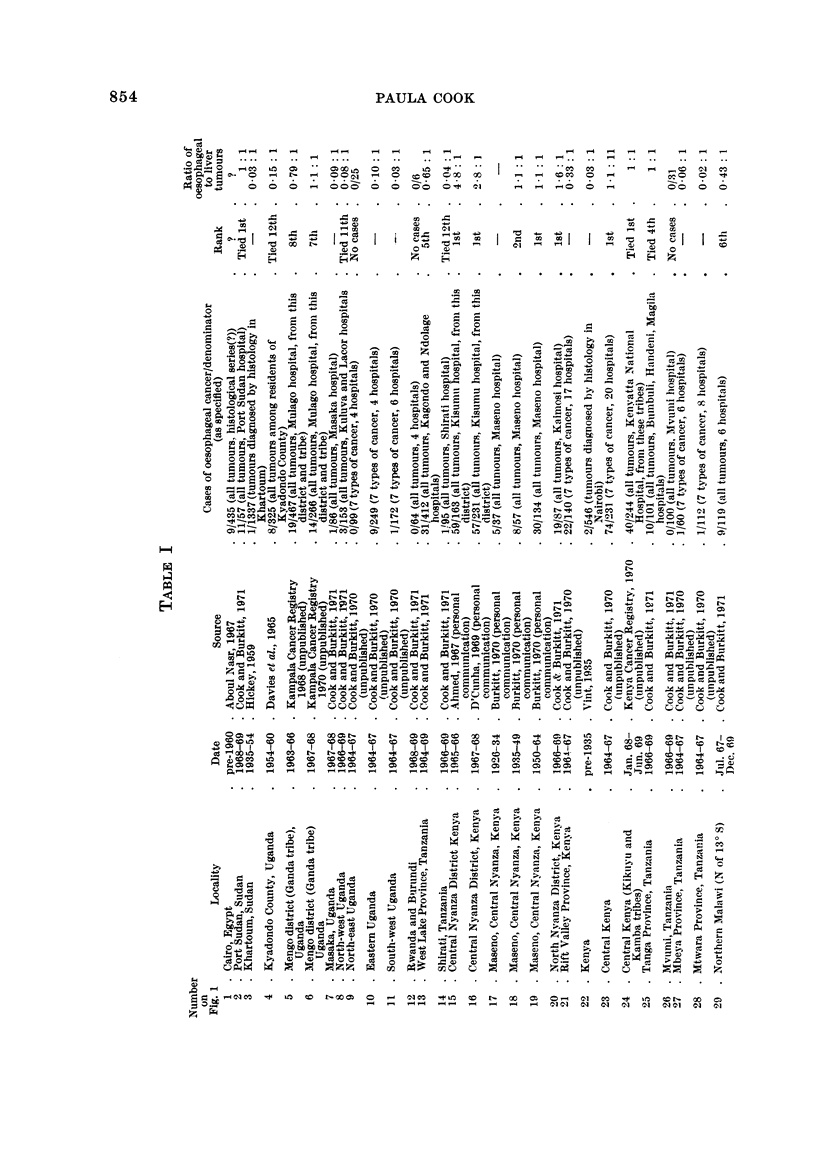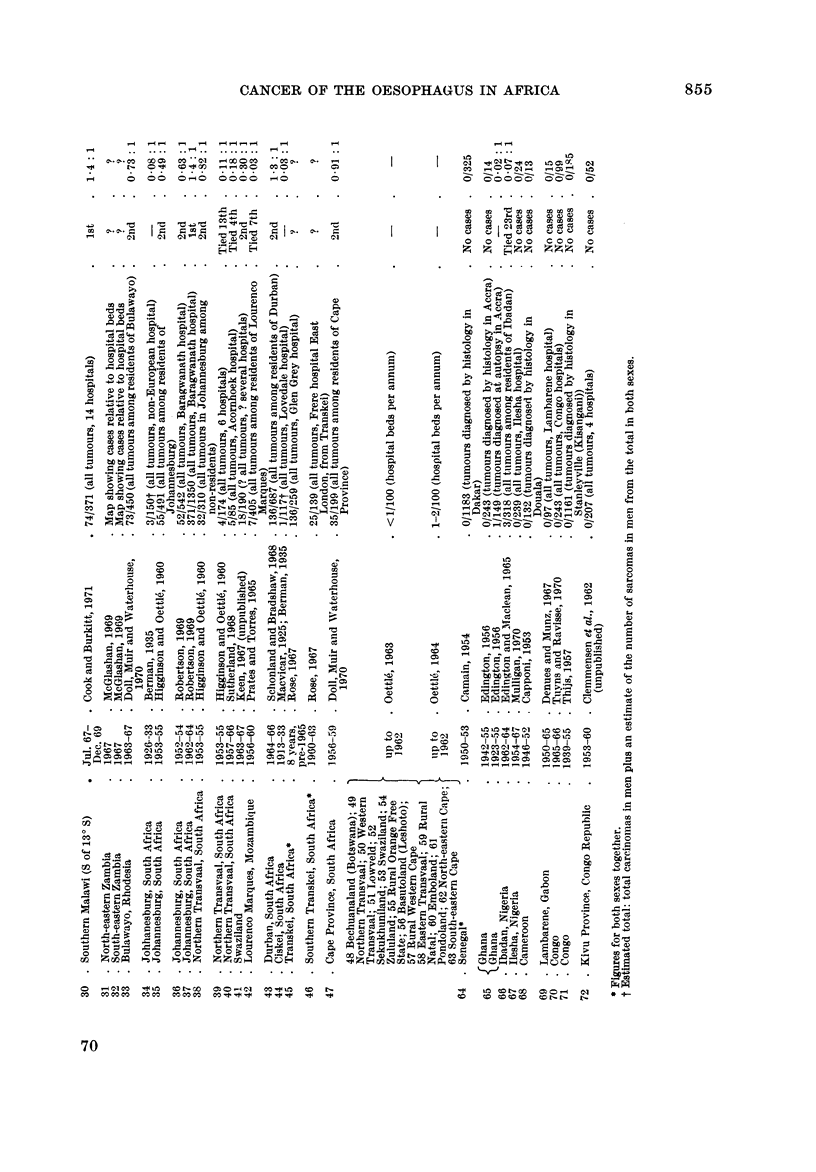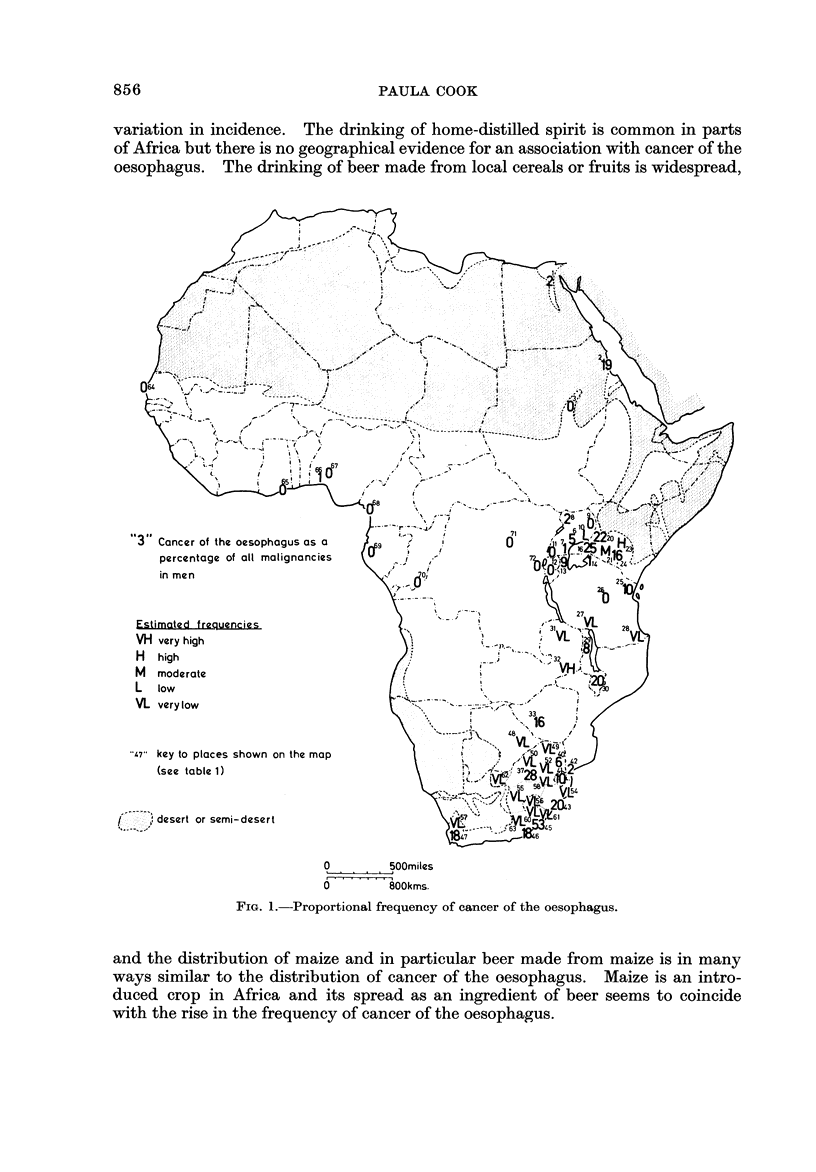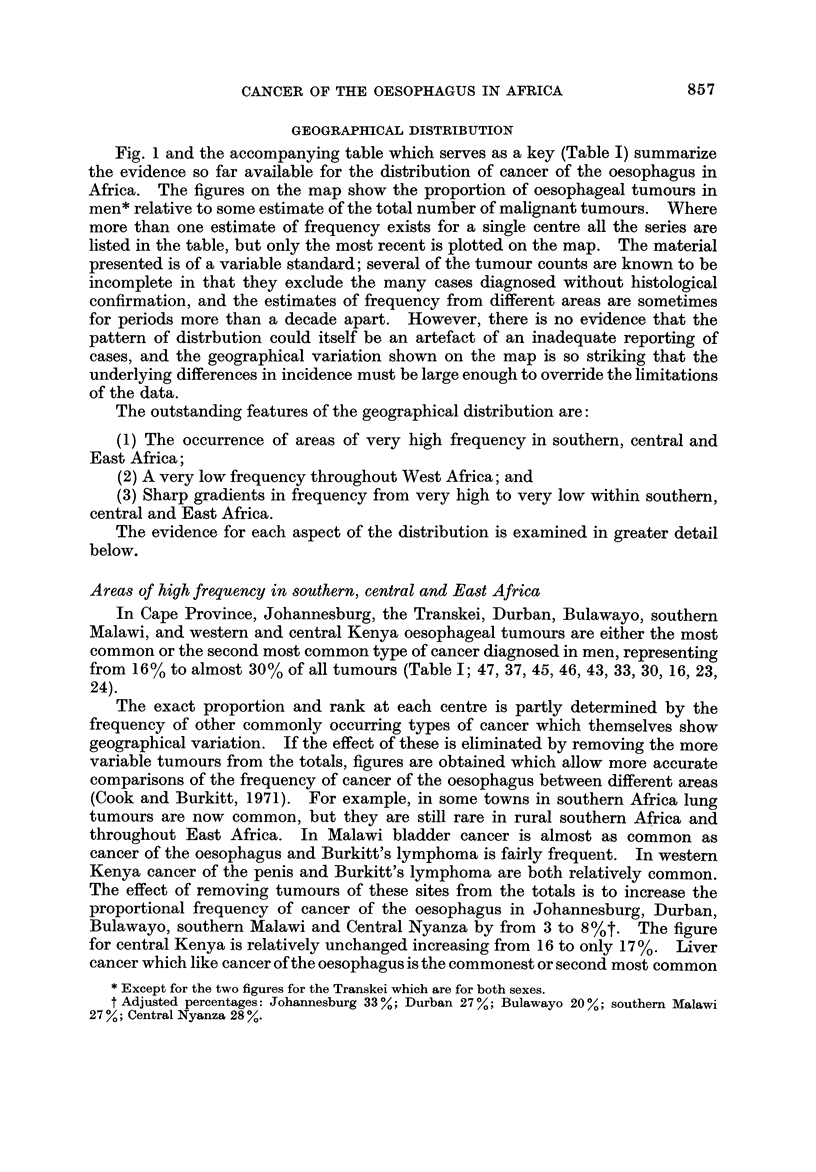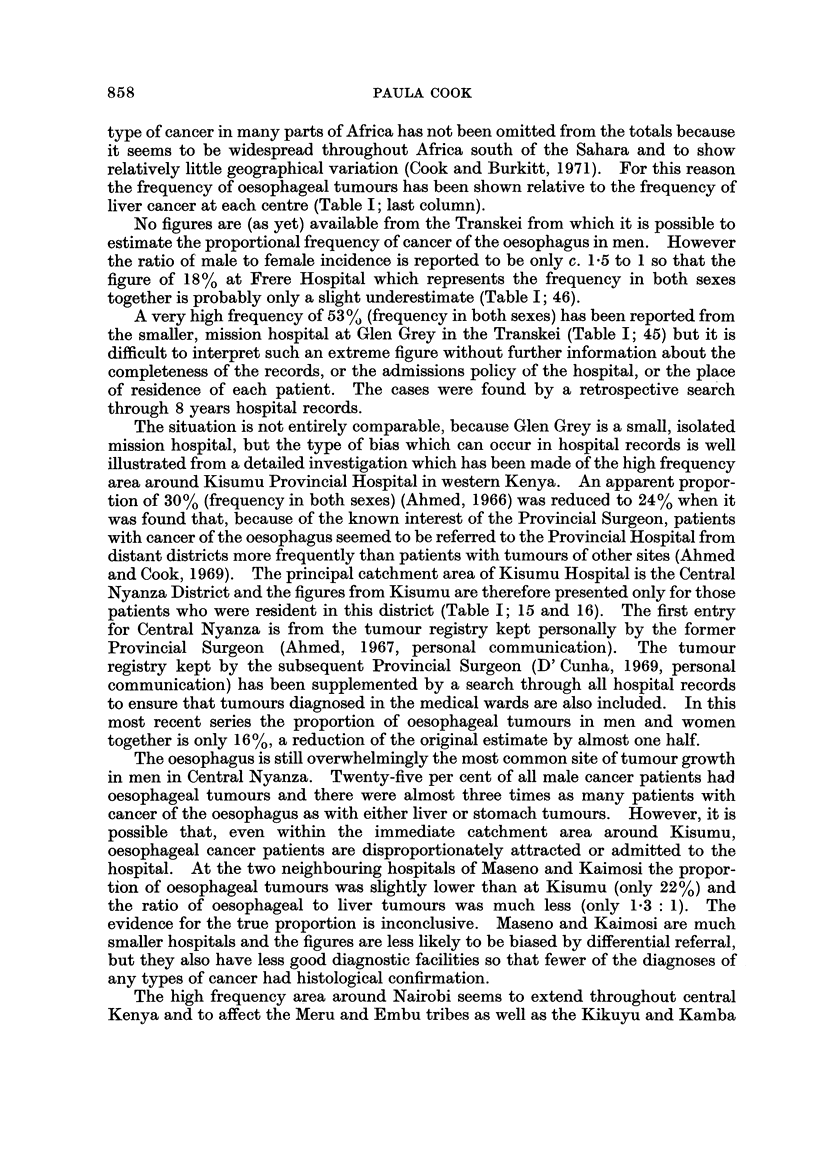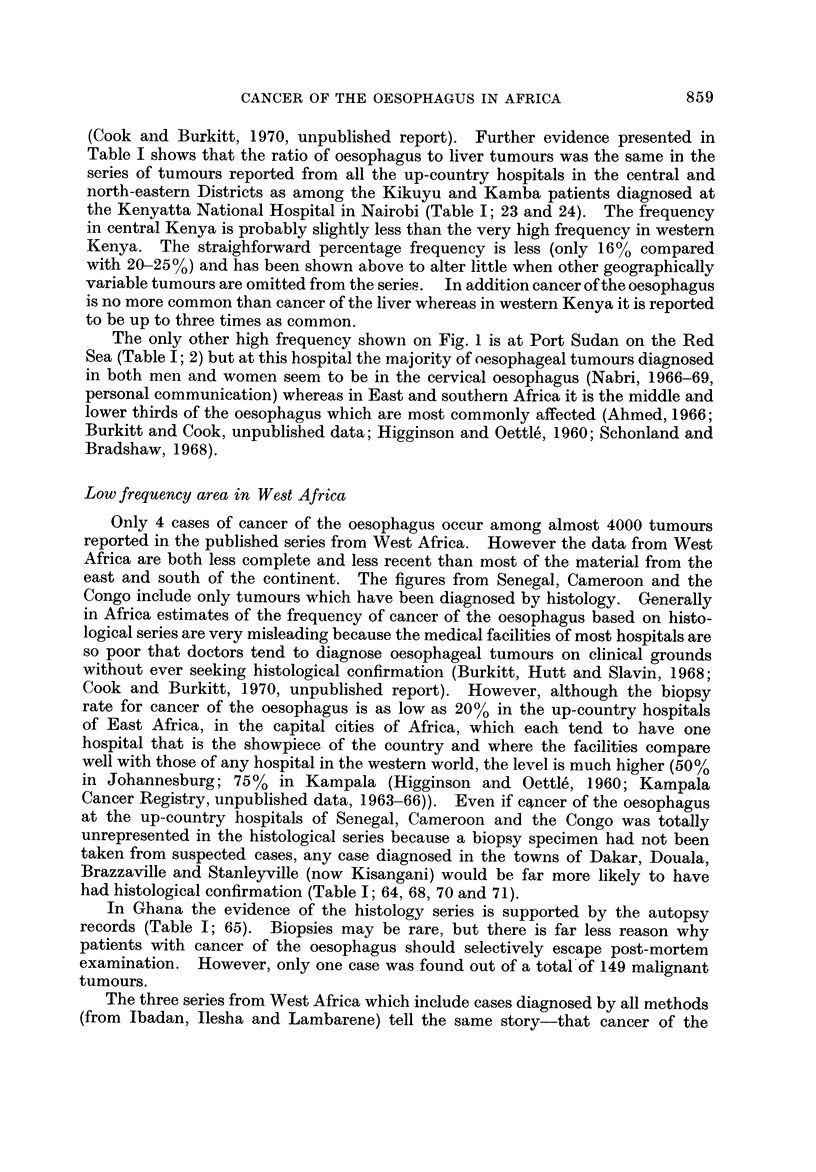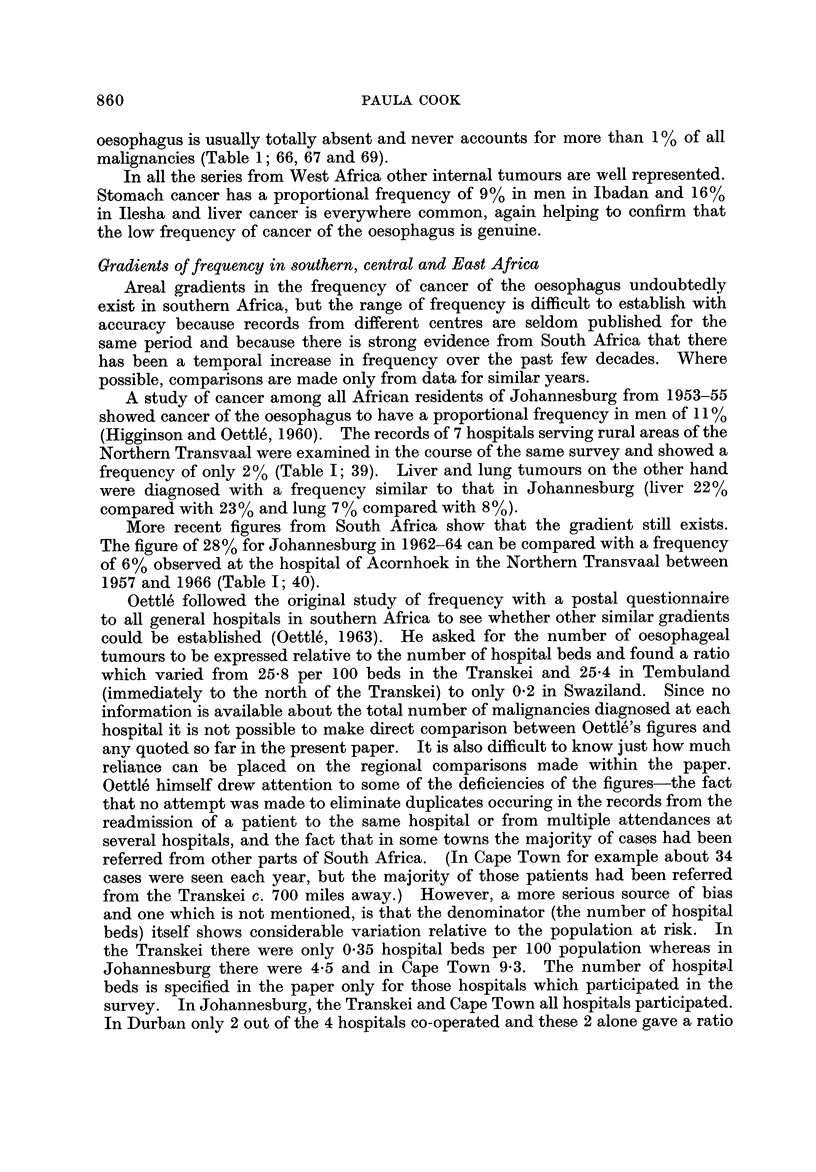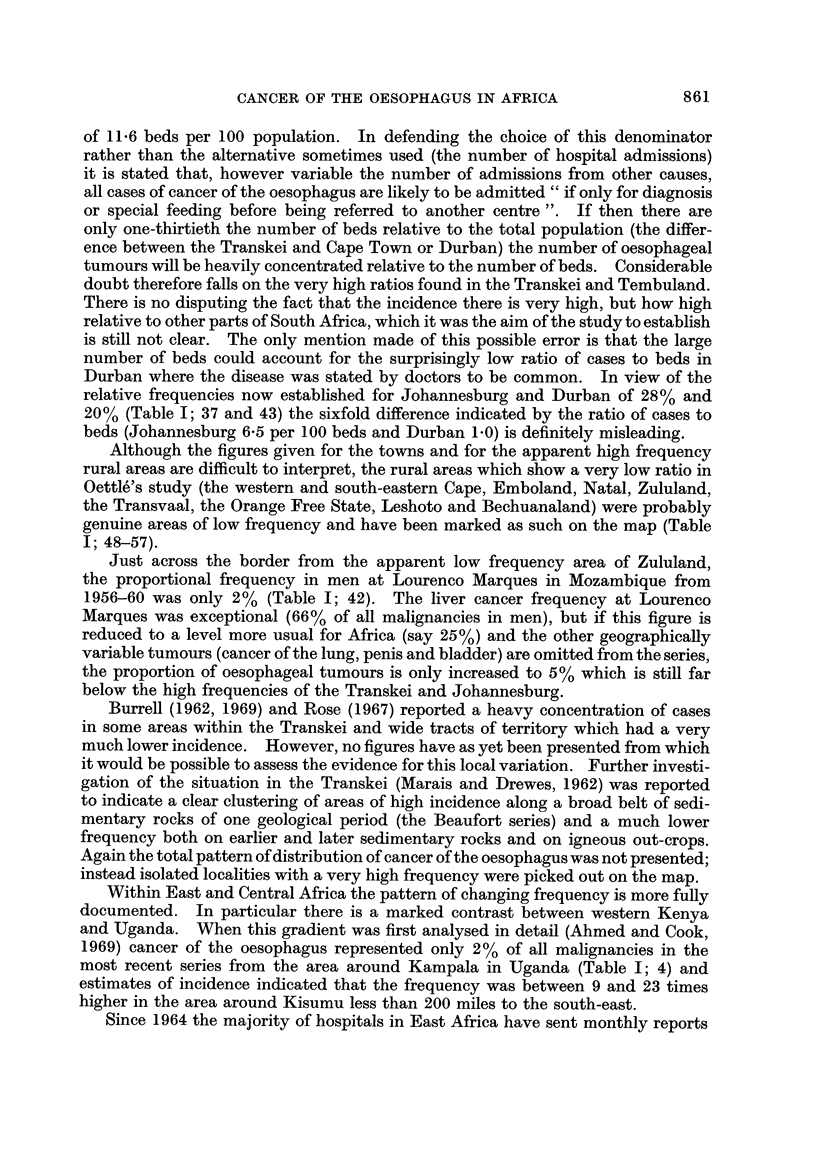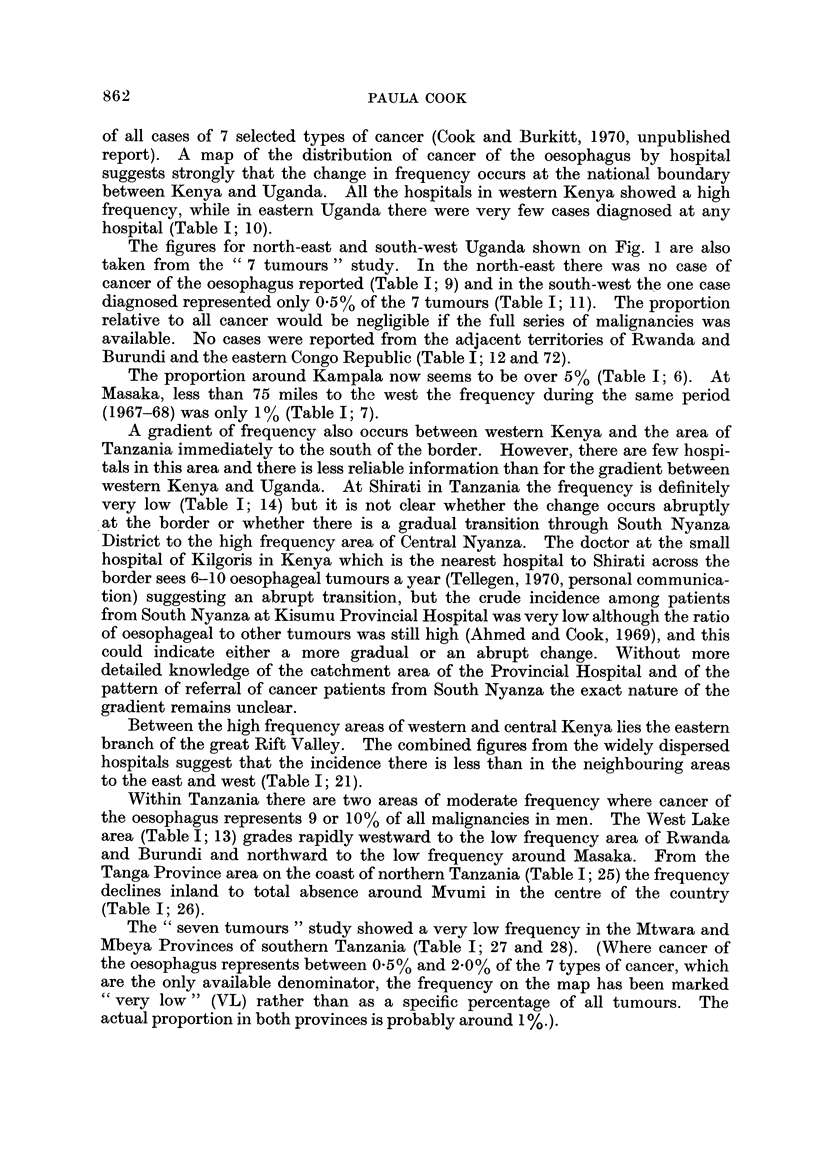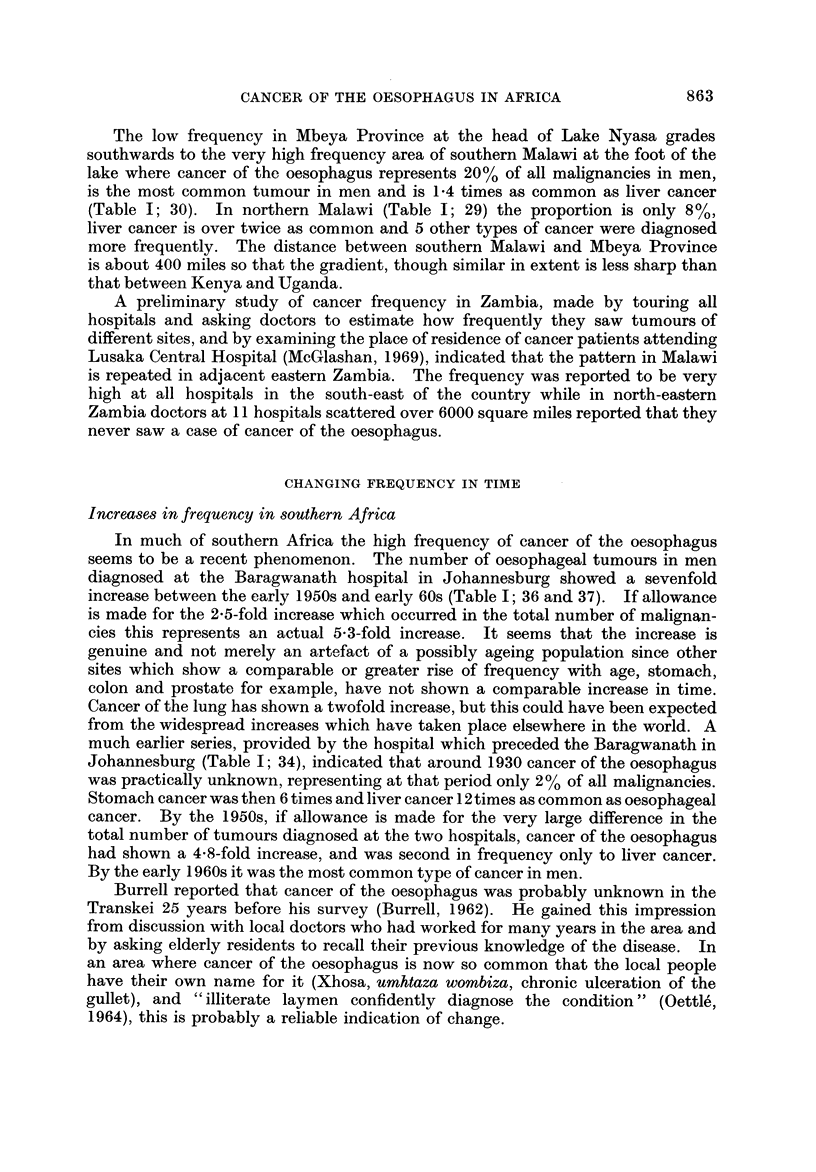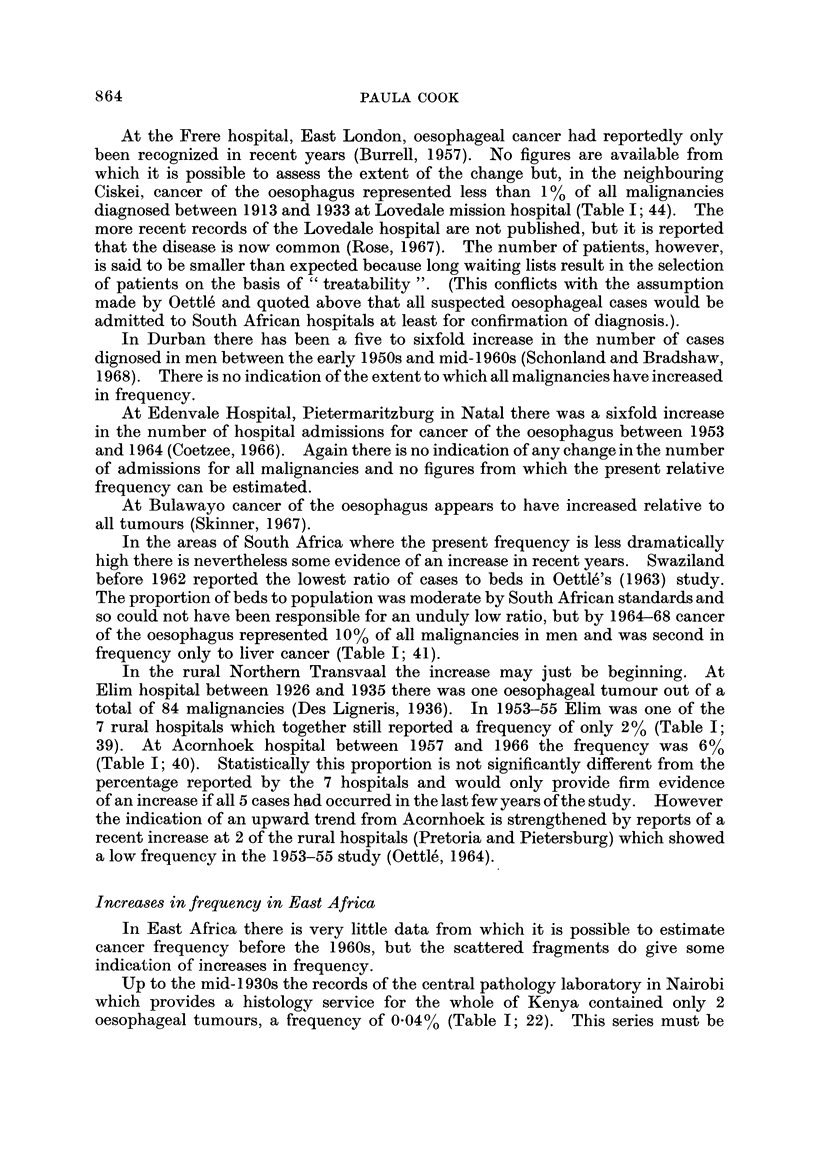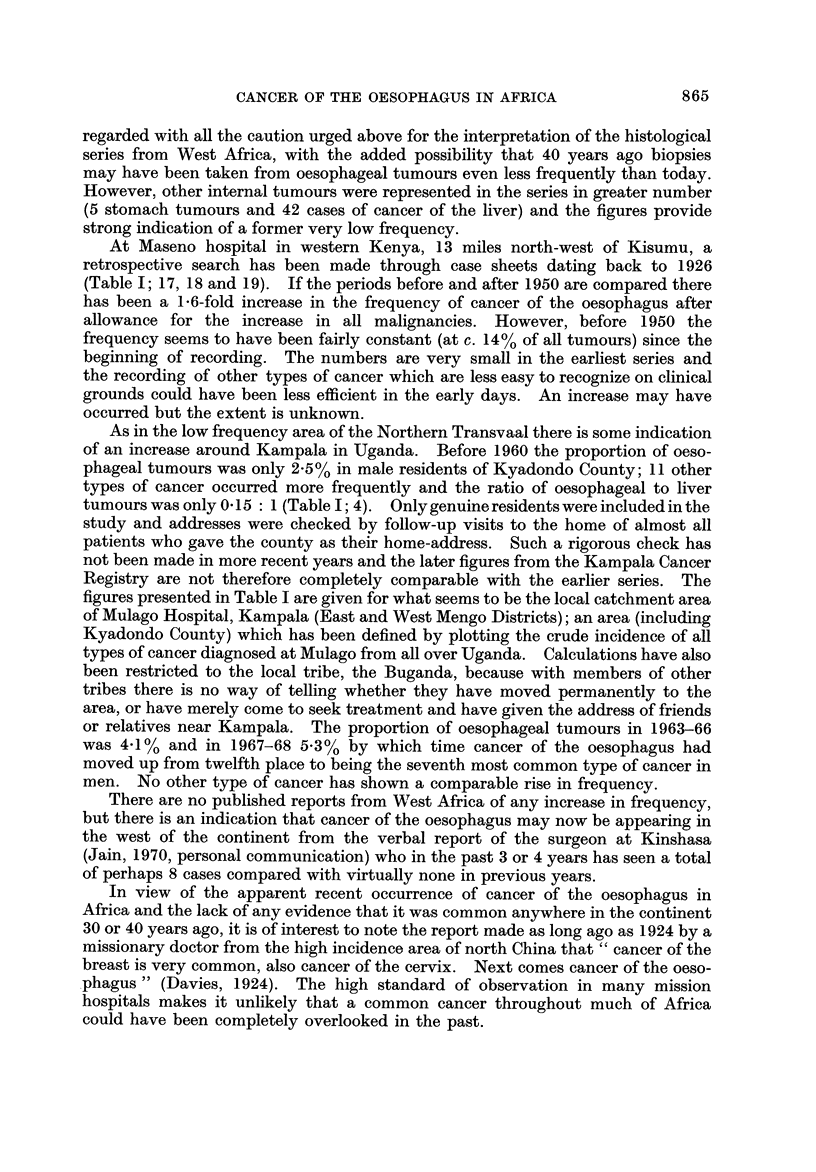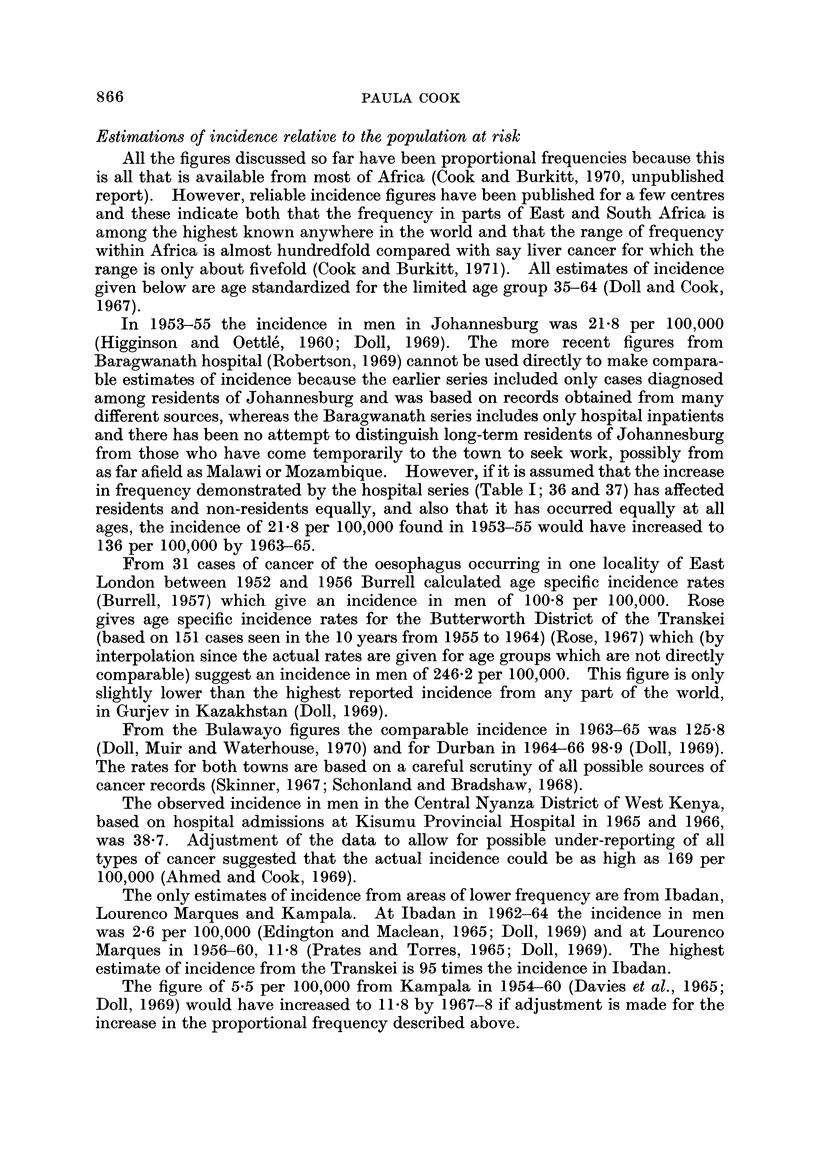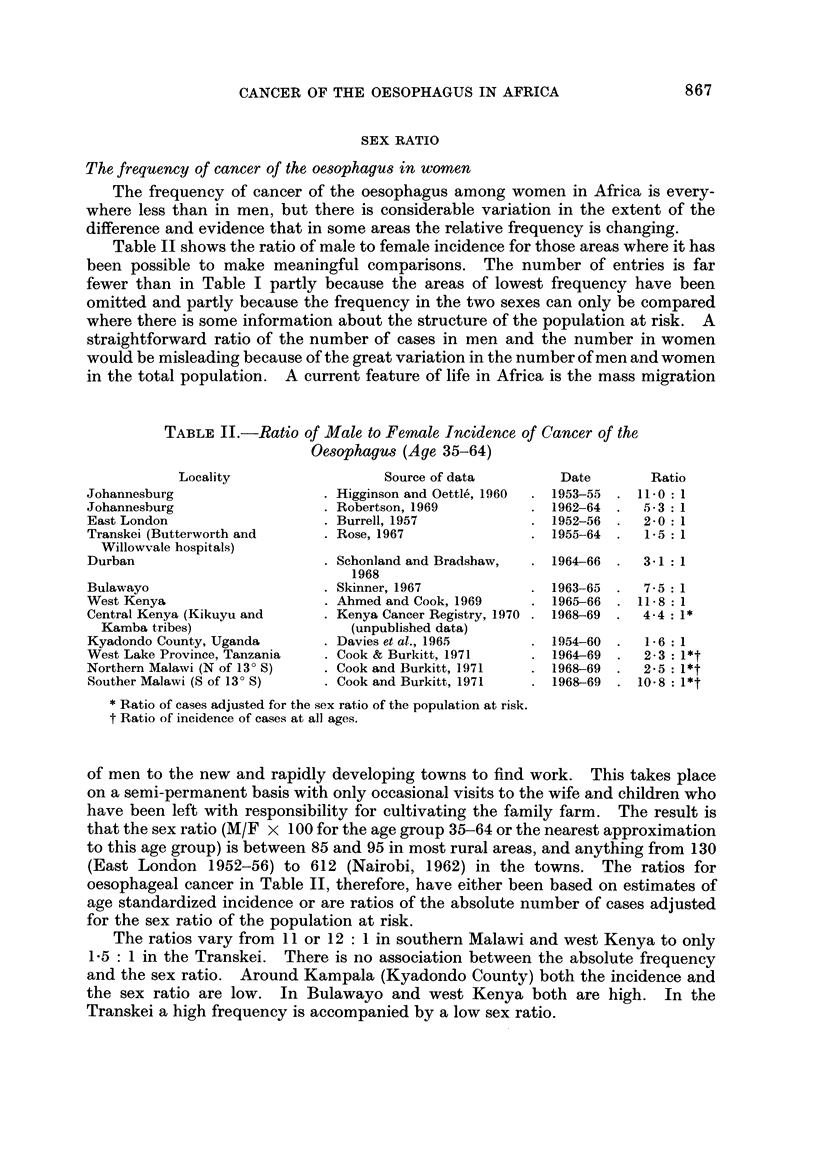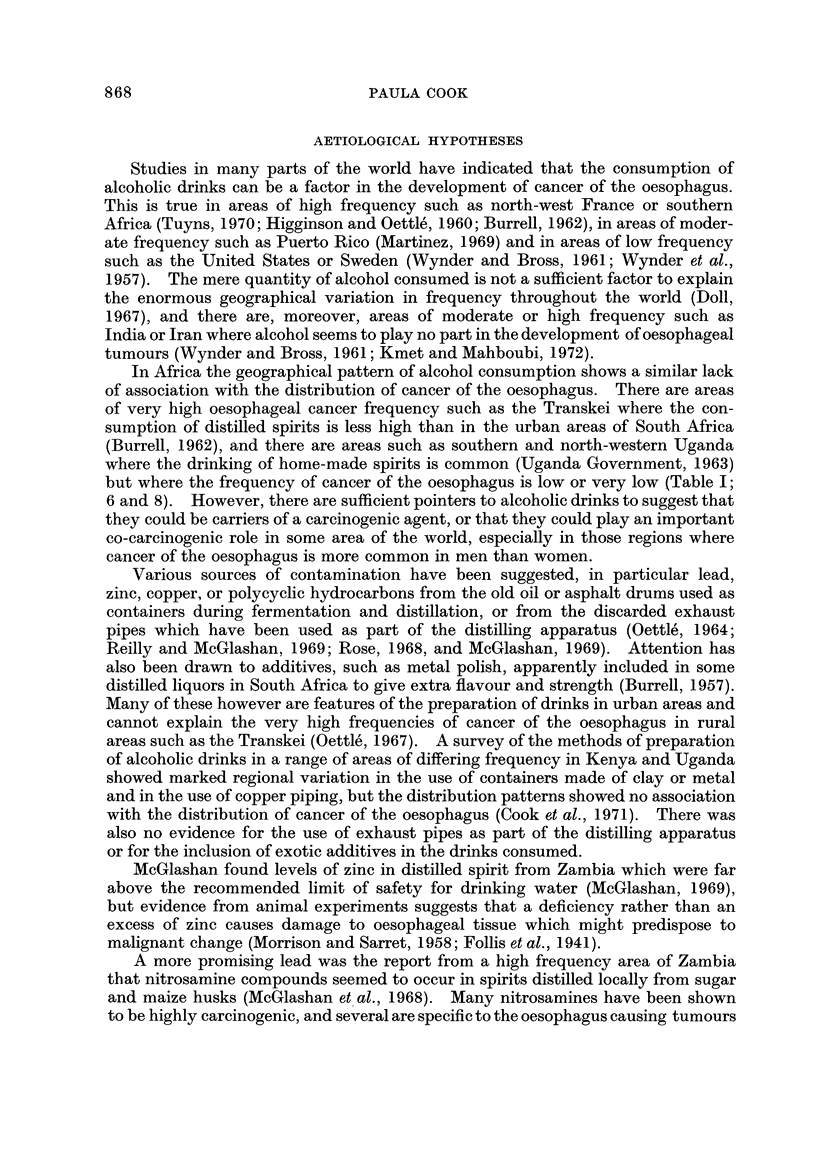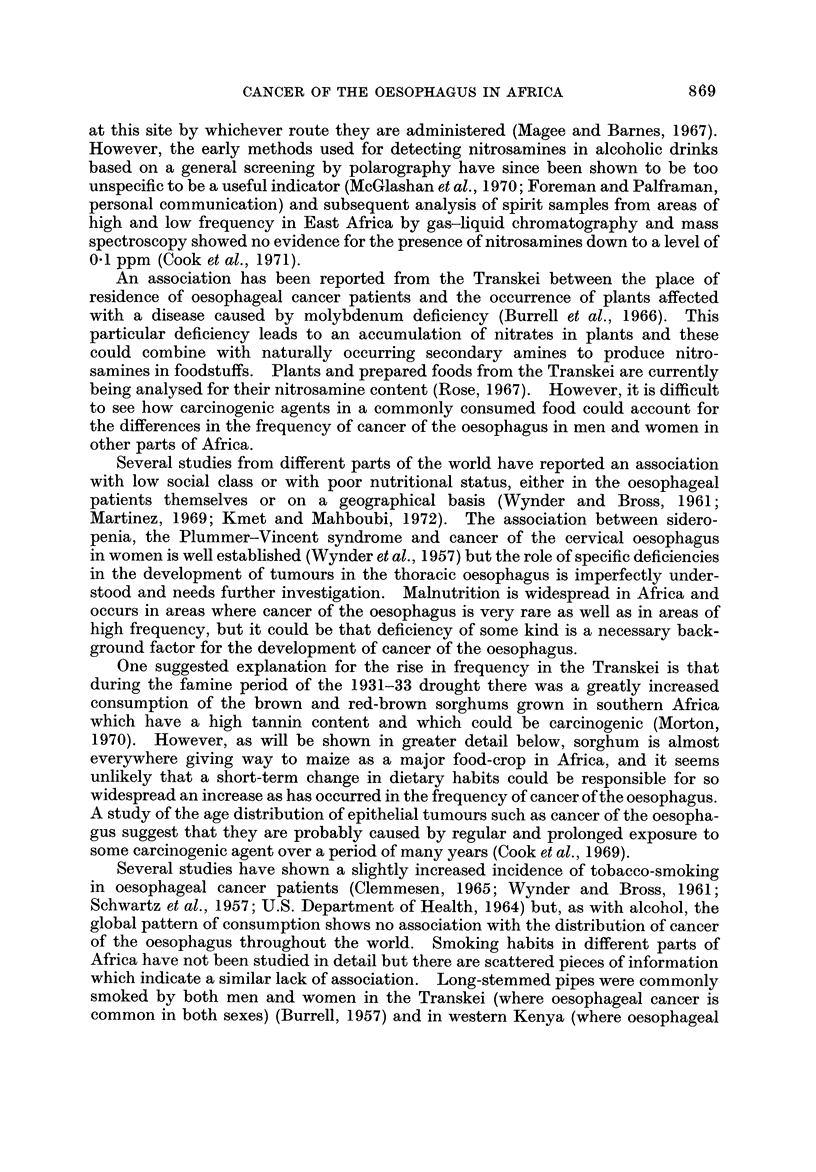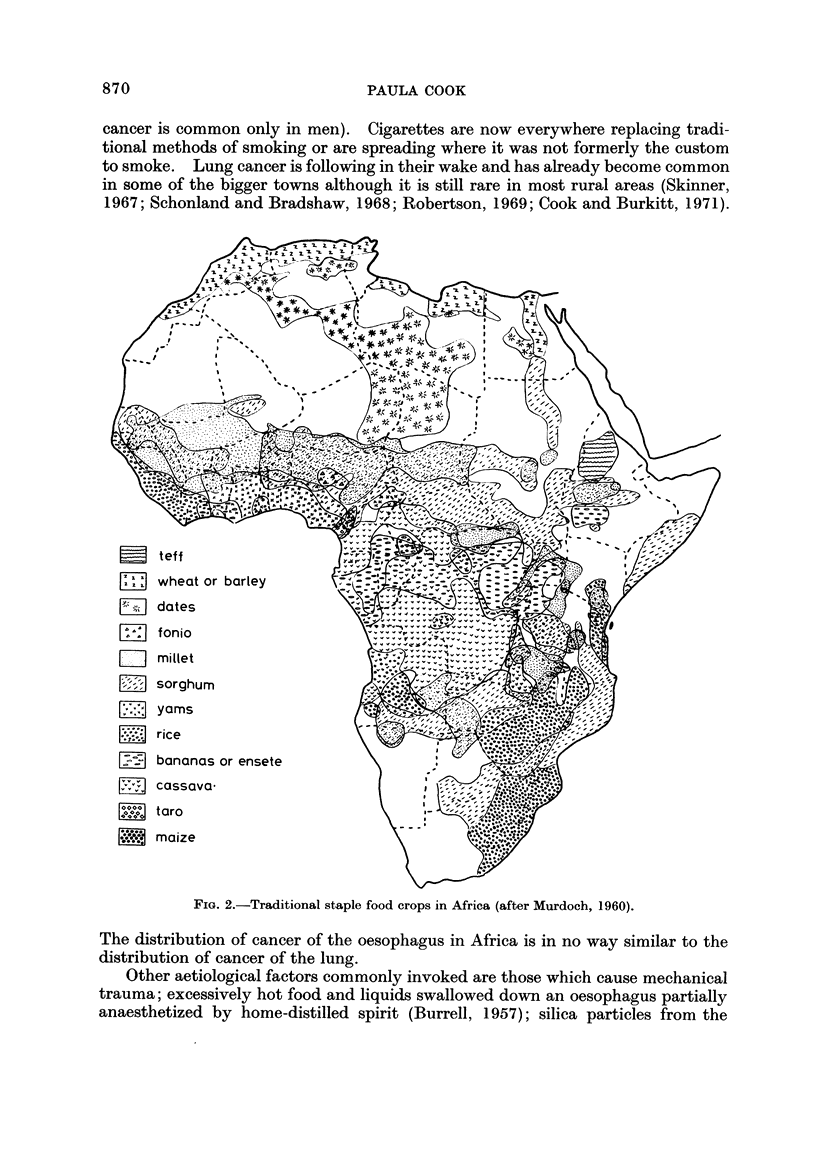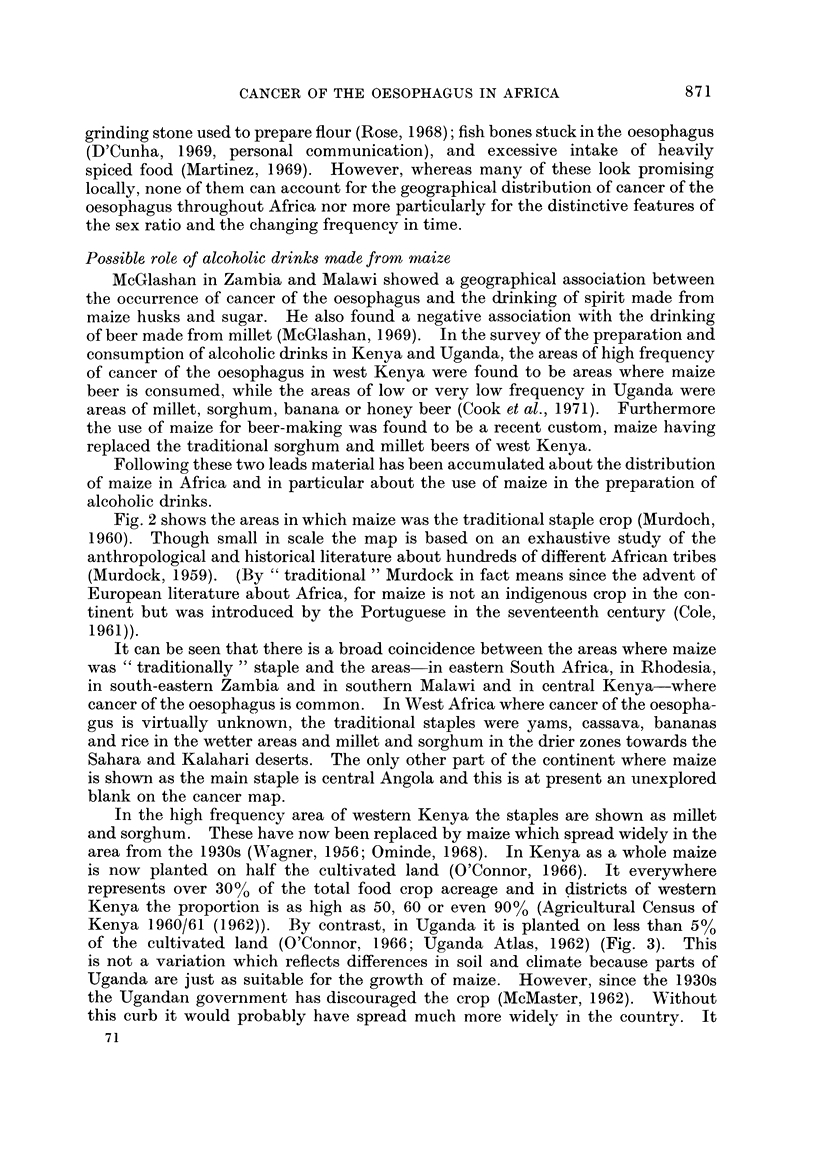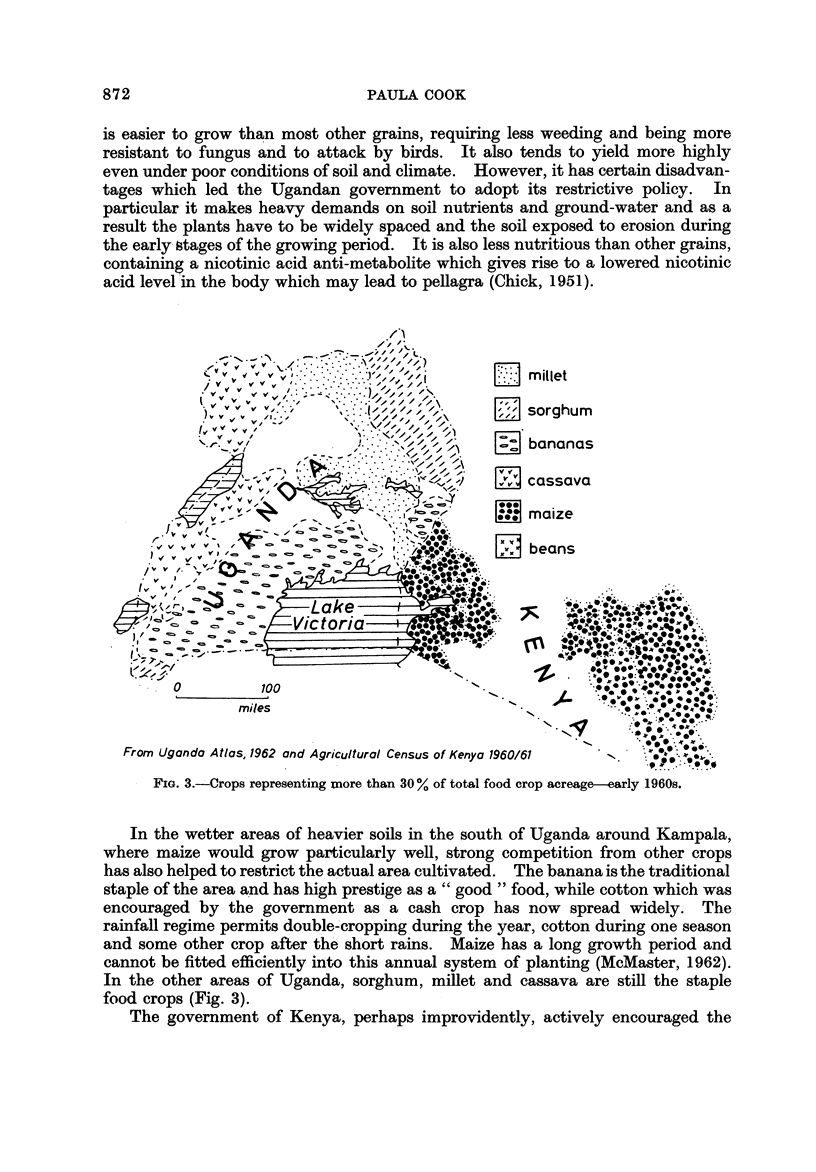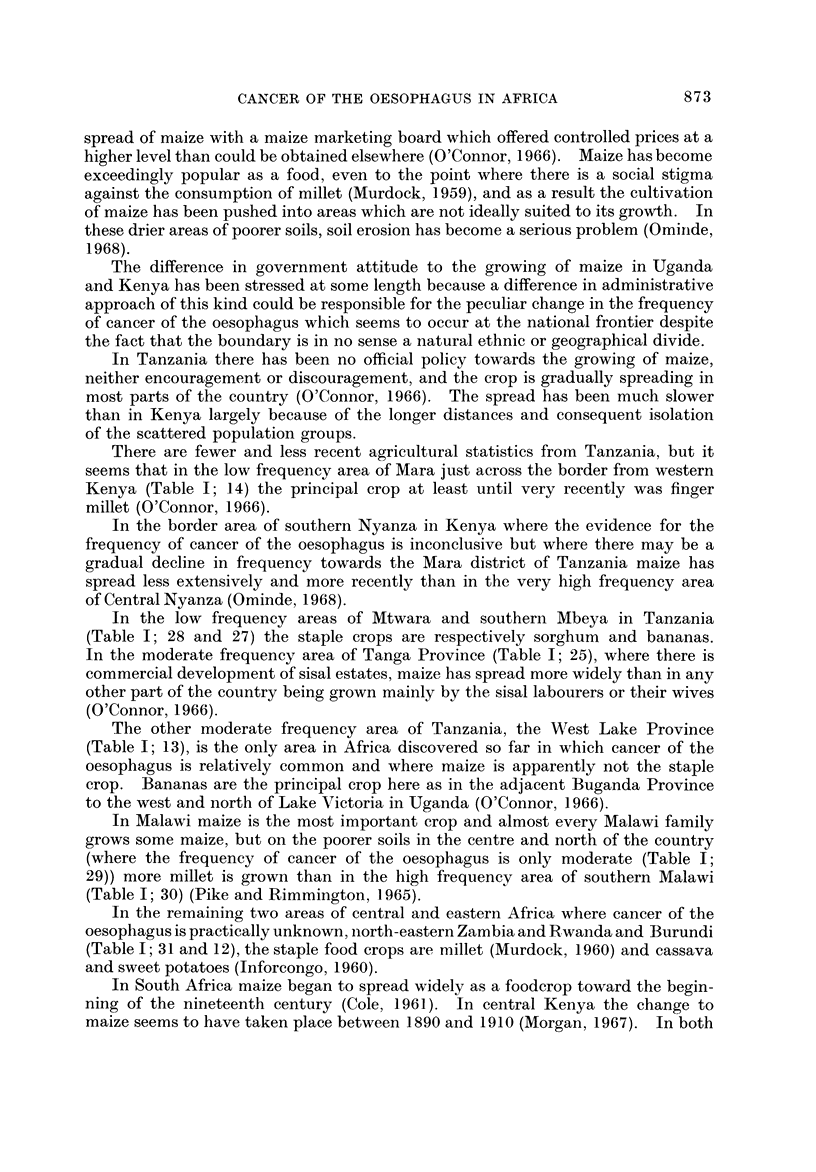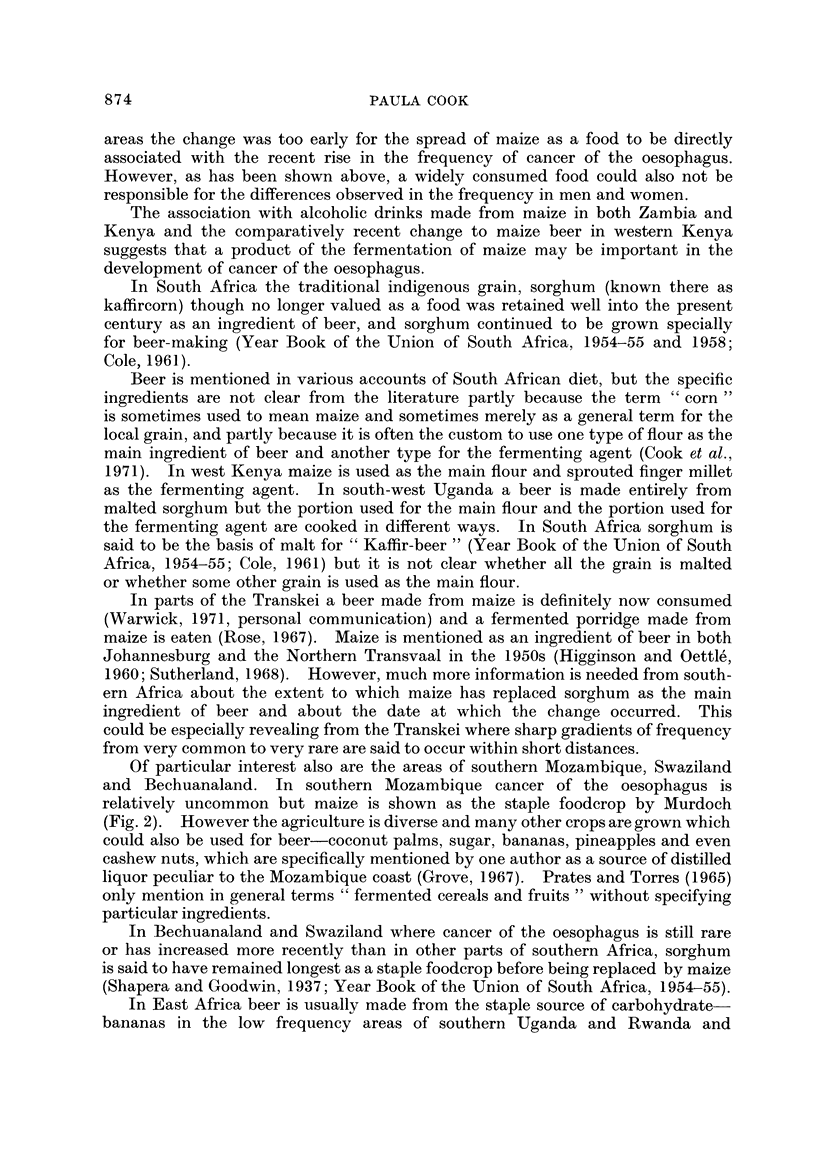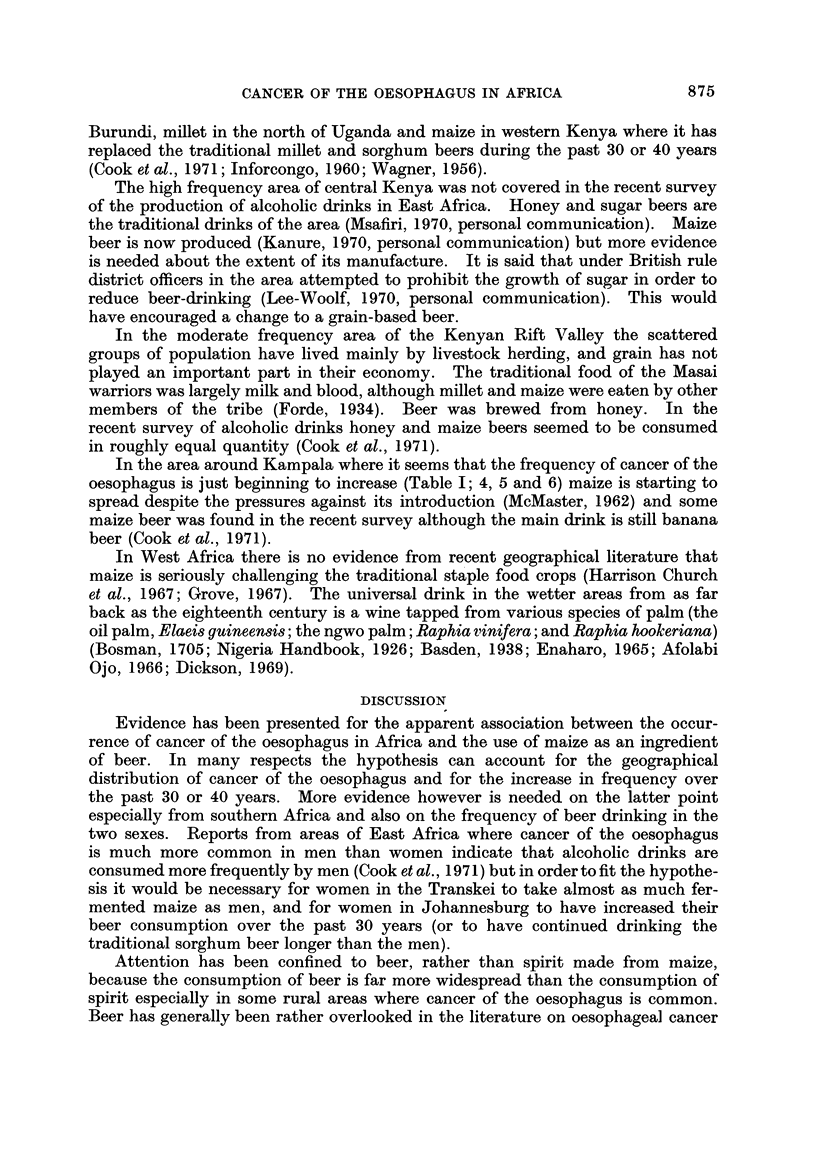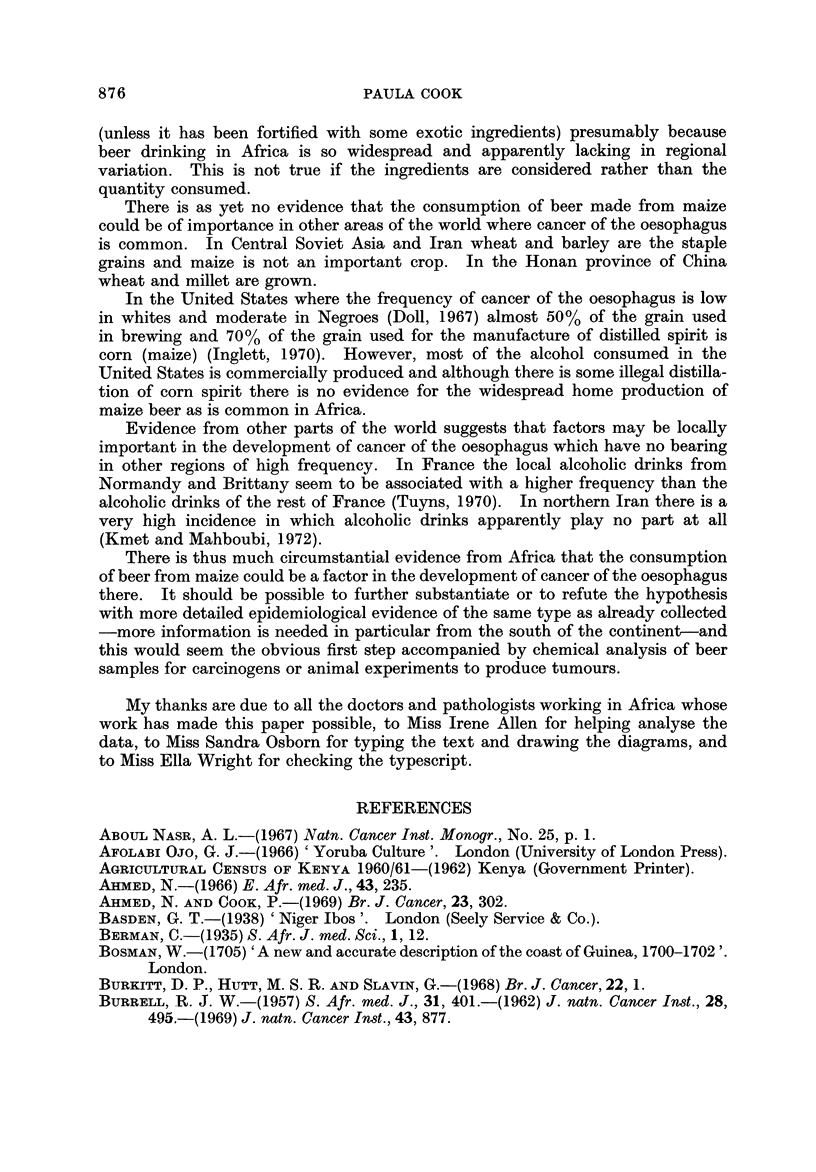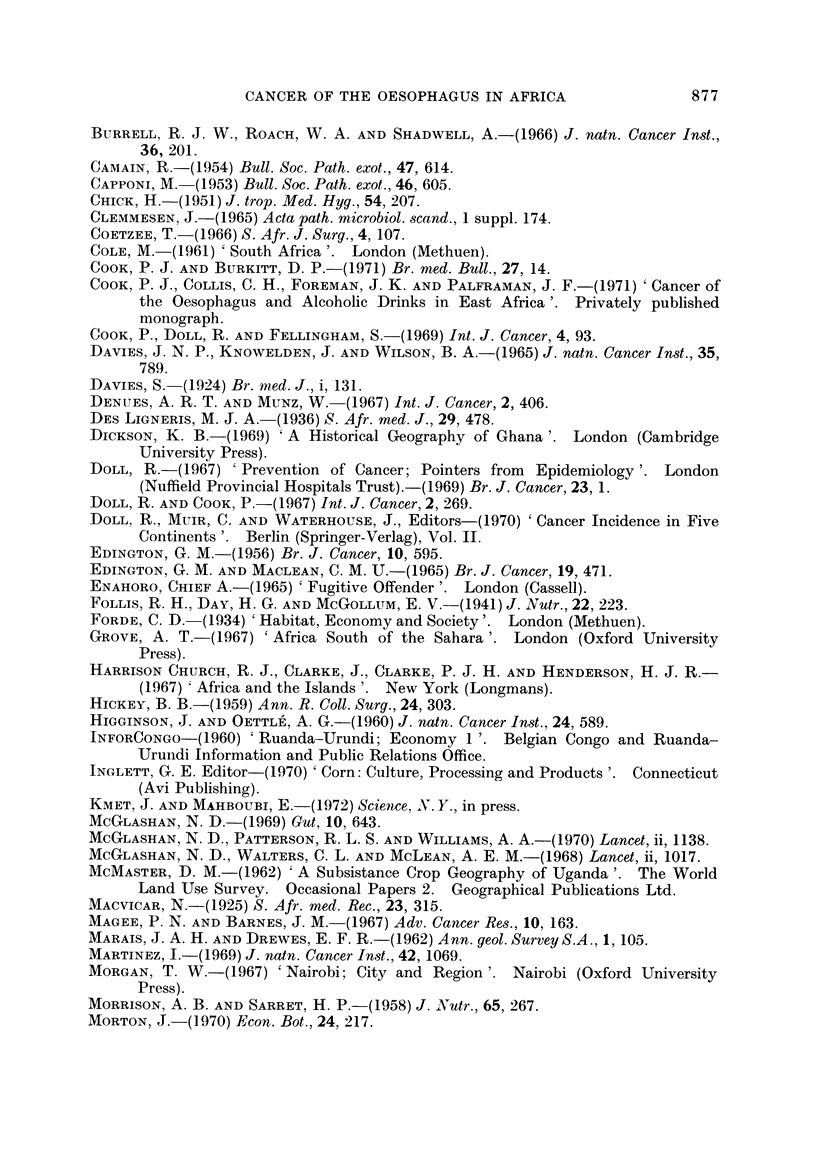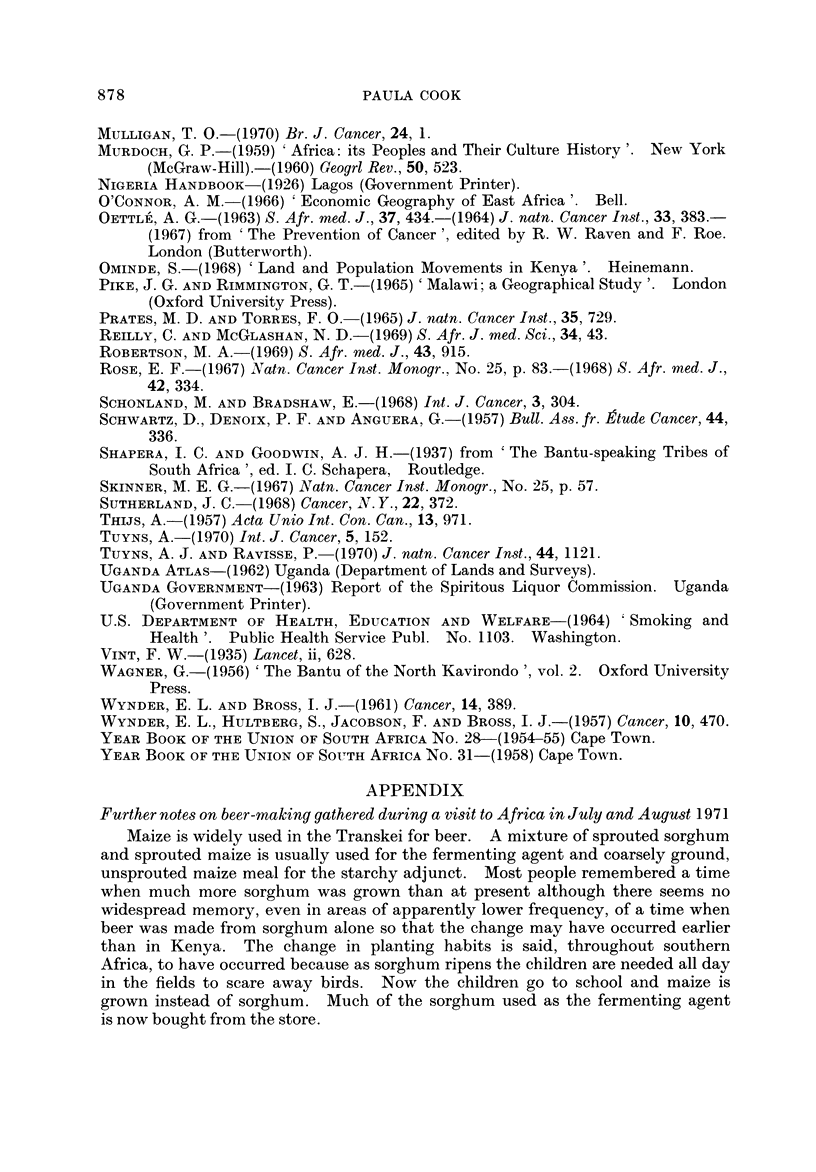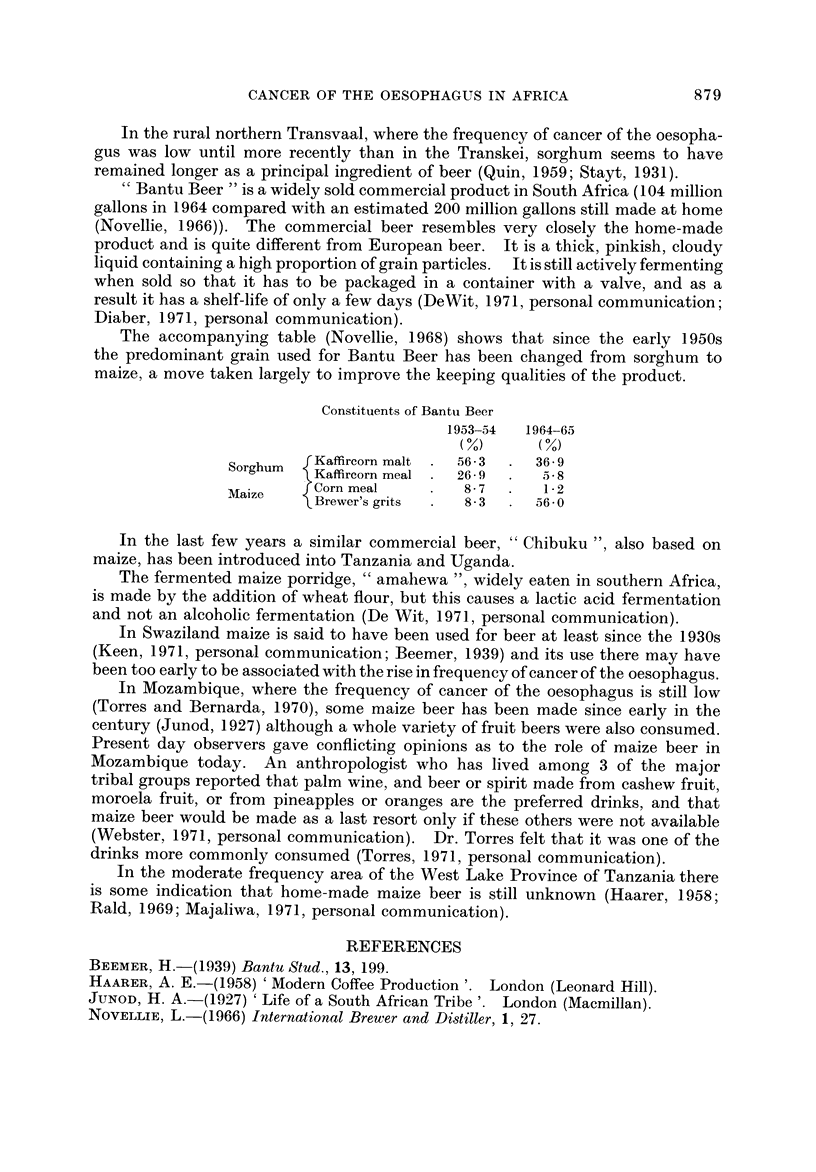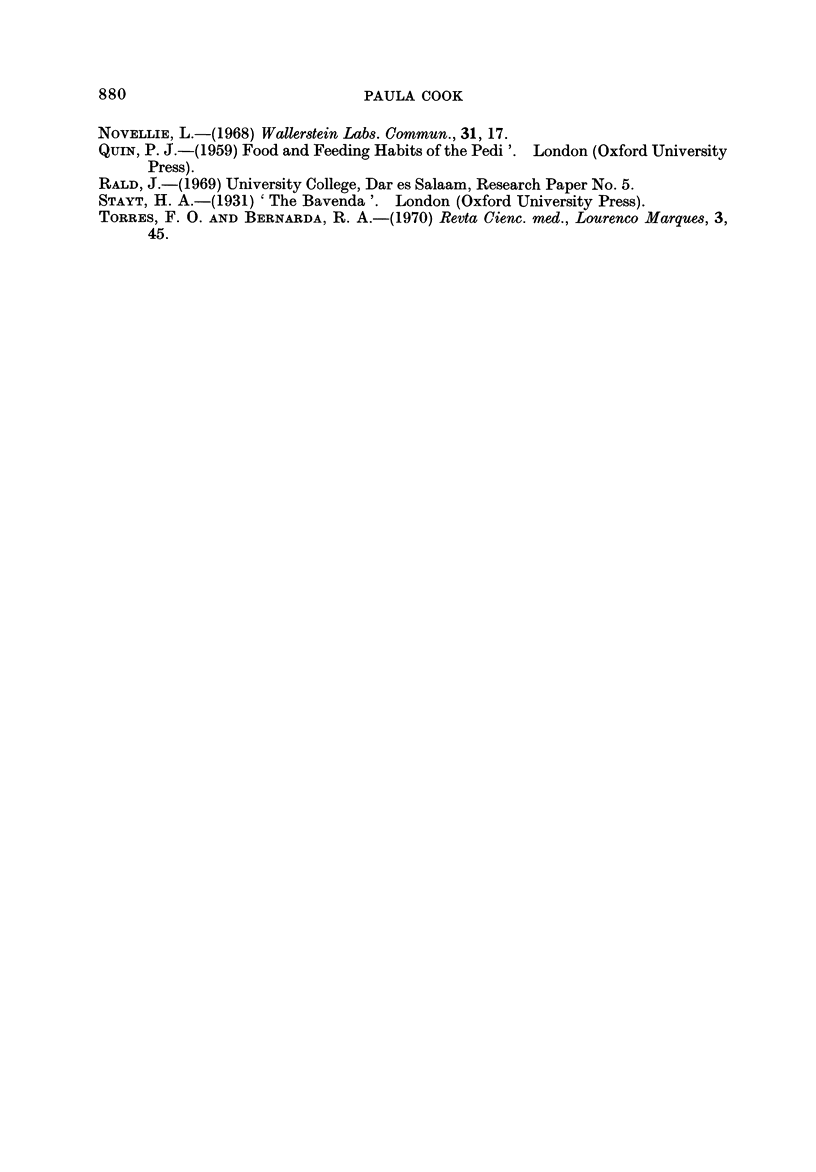# Cancer of the Oesophagus in Africa

**DOI:** 10.1038/bjc.1971.99

**Published:** 1971-12

**Authors:** Paula Cook

## Abstract

The oesophagus is the most common site of tumour development in men in parts of eastern and southern Africa. In West Africa cancer of the oesophagus is practically unknown. In the areas where it is common, the frequency is not uniformly high but shows sharp gradients within short distances. Most of the present high frequencies seem to have developed from a negligible incidence 30 or forty years ago. In all areas the disease is more common in men than women but the sex ratio varies from less than 2: 1 to 12: 1. Alcohol has been shown to be implicated in the development of cancer of the oesophagus elsewhere in the world. Home-made beer and spirit are common in many parts of Africa but there is no geographical association between frequency of consumption and the occurrence of oesophageal cancer. Evidence exists which suggests that both the geographical and temporal distributions in Africa could reflect the use of maize as a major ingredient of alcoholic drinks.


### CANCER OF THE OESOPHAGUS IN AFRICA

### A SUMMARY AND EVALUATION OF THE EVIDENCE FOR THE FREQUENCY OF OCCURRENCE, AND A PRELIMINARY INDICATION OF THE POSSIBLE ASSOCIATION WITH THE CONSUMPTION OF ALCOHOLIC DRINKS MADE FROM MAIZE

PAULA COOK

*From the M.R.C. Statistical Research and Services Unit, 115 Gower Street, London, W.C.1*

Received for publication September 14, 1971

**SUMMARY.—The oesophagus is the most common site of tumour development in men in parts of eastern and southern Africa. In West Africa cancer of the oesophagus is practically unknown. In the areas where it is common, the frequency is not uniformly high but shows sharp gradients within short distances. Most of the present high frequencies seem to have developed from a negligible incidence 30 or forty years ago. In all areas the disease is more common in men than women but the sex ratio varies from less than 2 : 1 to 12 : 1. Alcohol has been shown to be implicated in the development of cancer of the oesophagus elsewhere in the world. Home-made beer and spirit are common in many parts of Africa but there is no geographical association between frequency of consumption and the occurrence of oesophageal cancer. Evidence exists which suggests that both the geographical and temporal distributions in Africa could reflect the use of maize as a major ingredient of alcoholic drinks.**

THERE are three marked peculiarities in the occurrence of cancer of the oesophagus in Africa. The first is the geographical distribution; the second the changing pattern of frequency with time, and the third, the vagaries of the sex ratio. The incidence in parts of East and South Africa is among the highest recorded anywhere in the world while in West Africa it is virtually unknown; within East, central and southern Africa there are very steep gradients of frequency such that in one area it is the most commonly diagnosed tumour in men while less than 100 miles away it is rarely seen. Most of the present high frequencies seem to have developed from a negligible incidence 30 or 40 years ago. In all areas cancer of the oesophagus is more common in men than in women but the sex ratio varies from 1·5 : 1 in the Transkei to 12 : 1 in west Kenya and there is no association between the sex ratio and the general level of incidence. The geographical distribution suggests strongly that some environmental factor is involved in the development of cancer of the oesophagus. The variation in the sex ratio could indicate that the relevant factor lies in the cultural rather than the physical or biological environments.

An association with alcohol consumption has been established elsewhere in the world but the mere quantity of alcohol consumed is insufficient to explain global

TABLE I

| Number on Fig. 1 | Locality | Date | Source | Cases of oesophageal cancer/denominator (as specified) | Rank | Ratio of oesophageal to liver tumours |
|---|---|---|---|---|---|---|
| 1 | Cairo, Egypt | pre-1960 | Aboul Nasr, 1967 | 9/435 (all tumours, histological series(?)) | ? | ? |
| 2 | Port Sudan, Sudan | 1968–69 | Cook and Burkitt, 1971 | 11/57 (all tumours, Port Sudan hospital) | Tied 1st | 1 : 1 |
| 3 | Khartoum, Sudan | 1935–54 | Hickey, 1959 | 1/1337 (tumours diagnosed by histology in Khartoum) | — | 0·03 : 1 |
| 4 | Kyadondo County, Uganda | 1954–60 | Davies *et al.*, 1965 | 8/325 (all tumours among residents of Kyadondo County) | Tied 12th | 0·15 : 1 |
| 5 | Mengo district (Ganda tribe), Uganda | 1963–66 | Kampala Cancer Registry 1968 (unpublished) | 19/467 (all tumours, Mulago hospital, from this district and tribe) | 8th | 0·79 : 1 |
| 6 | Mengo district (Ganda tribe) Uganda | 1967–68 | Kampala Cancer Registry 1970 (unpublished) | 14/266 (all tumours, Mulago hospital, from this district and tribe) | 7th | 1·1 : 1 |
| 7 | Masaka, Uganda | 1967–68 | Cook and Burkitt, 1971 | 1/86 (all tumours, Masaka hospital) | — | 0·09 : 1 |
| 8 | North-west Uganda | 1966–69 | Cook and Burkitt, 1971 | 3/153 (all tumours, Kuluva and Lacor hospitals | Tied 11th | 0·08 : 1 |
| 9 | North-east Uganda | 1964–67 | Cook and Burkitt, 1970 (unpublished) | 0/99 (7 types of cancer, 4 hospitals) | No cases | 0/25 |
| 10 | Eastern Uganda | 1964–67 | Cook and Burkitt, 1970 (unpublished) | 9/249 (7 types of cancer, 4 hospitals) | — | 0·10 : 1 |
| 11 | South-west Uganda | 1964–67 | Cook and Burkitt, 1970 (unpublished) | 1/172 (7 types of cancer, 6 hospitals) | — | 0·03 : 1 |
| 12 | Rwanda and Burundi | 1968–69 | Cook and Burkitt, 1971 | 0/64 (all tumours, 4 hospitals) | No cases | 0/6 |
| 13 | West Lake Province, Tanzania | 1964–69 | Cook and Burkitt, 1971 | 31/412 (all tumours, Kagondo and Ndolage hospitals) | 5th | 0·65 : 1 |
| 14 | Shirati, Tanzania | 1966–69 | Cook and Burkitt, 1971 | 1/95 (all tumours, Shirati hospital) | Tied 12th | 0·04 : 1 |
| 15 | Central Nyanza District Kenya | 1965–66 | Ahmed, 1967 (personal communication) | 59/163 (all tumours, Kisumu hospital, from this district) | 1st | 4·8 : 1 |
| 16 | Central Nyanza District, Kenya | 1967–68 | D'Cunha, 1969 (personal communication) | 57/231 (all tumours, Kisumu hospital, from this district) | 1st | 2·8 : 1 |
| 17 | Maseno, Central Nyanza, Kenya | 1926–34 | Burkitt, 1970 (personal communication) | 5/37 (all tumours, Maseno hospital) | — | — |
| 18 | Maseno, Central Nyanza, Kenya | 1935–49 | Burkitt, 1970 (personal communication) | 8/57 (all tumours, Maseno hospital) | 2nd | 1·1 : 1 |
| 19 | Maseno, Central Nyanza, Kenya | 1950–64 | Burkitt, 1970 (personal communication) | 30/134 (all tumours, Maseno hospital) | 1st | 1·1 : 1 |
| 20 | North Nyanza District, Kenya | 1966–69 | Cook & Burkitt, 1971 | 19/87 (all tumours, Kaimosi hospital) | 1st | 1·6 : 1 |
| 21 | Rift Valley Province, Kenya | 1964–67 | Cook and Burkitt, 1970 (unpublished) | 22/140 (7 types of cancer, 17 hospitals) | — | 0·33 : 1 |
| 22 | Kenya | pre-1935 | Vint, 1935 | 2/546 (tumours diagnosed by histology in Nairobi) | — | 0·03 : 1 |
| 23 | Central Kenya | 1964–67 | Cook and Burkitt, 1970 (unpublished) | 74/231 (7 types of cancer, 20 hospitals) | 1st | 1·1 : 11 |
| 24 | Central Kenya (Kikuyu and Kamba tribes) | Jan. 68–Jun. 69 | Kenya Cancer Registry, 1970 (unpublished) | 40/244 (all tumours, Kenyatta National Hospital, from these tribes) | Tied 1st | 1 : 1 |
| 25 | Tanga Province, Tanzania | 1966–69 | Cook and Burkitt, 1971 | 10/101 (all tumours, Bumbuli, Handeni, Magila hospitals) | Tied 4th | 1 : 1 |
| 26 | Mvumi, Tanzania | 1966–69 | Cook and Burkitt, 1971 | 0/100 (all tumours, Mvumi hospital) | No cases | 0/31 |
| 27 | Mbeya Province, Tanzania | 1964–67 | Cook and Burkitt, 1970 (unpublished) | 1/60 (7 types of cancer, 6 hospitals) | — | 0·06 : 1 |
| 28 | Mtwara Province, Tanzania | 1964–67 | Cook and Burkitt, 1970 (unpublished) | 1/112 (7 types of cancer, 8 hospitals) | — | 0·02 : 1 |
| 29 | Northern Malawi (N of 13° S) | Jul. 67–Dec. 69 | Cook and Burkitt, 1971 | 9/119 (all tumours, 6 hospitals) | 6th | 0·43 : 1 |

| | | | | | | |
|---|---|---|---|---|---|---|
| 30 | Southern Malawi (S of 13° S) | Jul. 67–Dec. 69 | Cook and Burkitt, 1971 | 74/371 (all tumours, 14 hospitals) | 1st | 1·4 : 1 |
| 31 | North-eastern Zambia | 1967 | McGlashan, 1969 | Map showing cases relative to hospital beds | ? | ? |
| 32 | South-eastern Zambia | 1967 | McGlashan, 1969 | Map showing cases relative to hospital beds | ? | ? |
| 33 | Bulawayo, Rhodesia | 1963–67 | Doll, Muir and Waterhouse, 1970 | 73/450 (all tumours among residents of Bulawayo) | 2nd | 0·73 : 1 |
| 34 | Johhanesburg, South Africa | 1926–33 | Berman, 1935 | 3/150† (all tumours, non-European hospital) | — | 0·08 : 1 |
| 35 | Johannesburg, South Africa | 1953–55 | Higginson and Oettlé, 1960 | 55/491 (all tumours among residents of Johannesburg) | 2nd | 0·49 : 1 |
| 36 | Johannesburg, South Africa | 1952–54 | Robertson, 1969 | 52/542 (all tumours, Baragwanath hospital) | 2nd | 0·63 : 1 |
| 37 | Johannesburg, South Africa | 1962–64 | Robertson, 1969 | 371/1350 (all tumours, Baragwanath hospital) | 1st | 1·4 : 1 |
| 38 | Northern Transvaal, South Africa | 1953–55 | Higginson and Oettlé, 1960 | 32/310 (all tumours in Johannesburg among non-residents) | 2nd | 0·82 : 1 |
| 39 | Northern Transvaal, South Africa | 1953–55 | Higginson and Oettlé, 1960 | 4/174 (all tumours, 6 hospitals) | Tied 13th | 0·11 : 1 |
| 40 | Northern Transvaal, South Africa | 1957–66 | Sutherland, 1968 | 5/85 (all tumours, Acornhoek hospital) | Tied 4th | 0·18 : 1 |
| 41 | Swaziland | 1963–67 | Keen, 1967 (unpublished) | 18/190 (? all tumours, ? several hospitals) | 2nd | 0·30 : 1 |
| 42 | Lourenco Marques, Mozambique | 1956–60 | Prates and Torres, 1965 | 7/405 (all tumours among residents of Lourenco Marques) | Tied 7th | 0·03 : 1 |
| 43 | Durban, South Africa | 1964–66 | Schonland and Bradshaw, 1968 | 136/687 (all tumours among residents of Durban) | 2nd | 1·3 : 1 |
| 44 | Ciskei, South Africa | 1913–33 | Macvicar, 1925; Berman, 1935 | 1/117† (all tumours, Lovedale hospital) | — | 0·03 : 1 |
| 45 | Transkei, South Africa* | 8 years, pre-1965 | Rose, 1967 | 136/259 (all tumours, Glen Grey hospital) | ? | ? |
| 46 | Southern Transkei, South Africa* | 1960–63 | Rose, 1967 | 25/139 (all tumours, Frere hospital East London, from Transkei) | ? | ? |
| 47 | Cape Province, South Africa | 1956–59 | Doll, Muir and Waterhouse, 1970 | 35/199 (all tumours among residents of Cape Province) | 2nd | 0·91 : 1 |
| | 48 Bechuanaland (Botswana); 49 Northern Transvaal; 50 Western Transvaal; 51 Lowveld; 52 Sekukhuniland; 53 Swaziland; 54 Zululand; 55 Rural Orange Free State; 56 Basutoland (Leshoto); 57 Rural Western Cape | up to 1962 | Oettlé, 1963 | <1/100 (hospital beds per annum) | — | — |
| | 58 Eastern Transvaal; 59 Rural Natal; 60 Emboland; 61 Pondoland; 62 North-eastern Cape; 63 South-eastern Cape | up to 1962 | Oettlé, 1964 | 1–2/100 (hospital beds per annum) | — | — |
| 64 | Senegal* | 1950–53 | Camain, 1954 | 0/1183 (tumours diagnosed by histology in Dakar) | No cases | 0/325 |
| 65 | Ghana | 1942–55 | Edington, 1956 | 0/243 (tumours diagnosed by histology in Accra) | No cases | 0/14 |
| 65 | Ghana | 1923–55 | Edington, 1956 | 1/149 (tumours diagnosed at autopsy in Accra) | — | 0·02 : 1 |
| 66 | Ibadan, Nigeria | 1962–64 | Edington and Maclean, 1965 | 3/318 (all tumours among residents of Ibadan) | Tied 23rd | 0·07 : 1 |
| 67 | Ilesha, Nigeria | 1954–67 | Mulligan, 1970 | 0/239 (all tumours, Ilesha hospital) | No cases | 0/24 |
| 68 | Cameroon | 1946–52 | Capponi, 1953 | 0/132 (tumours diagnosed by histology in Douala) | No cases | 0/13 |
| 69 | Lambarene, Gabon | 1950–65 | Denues and Munz, 1967 | 0/97 (all tumours, Lambarene hospital) | No cases | 0/15 |
| 70 | Congo | 1965–66 | Tuyns and Ravisse, 1970 | 0/243 (all tumours, Congo hospitals) | No cases | 0/99 |
| 71 | Congo | 1939–55 | Thijs, 1957 | 0/1161 (tumours diagnosed by histology in Stanleyville (Kisangani)) | No cases | 0/185 |
| 72 | Kivu Province, Congo Republic | 1953–60 | Clemmensen *et al.*, 1962 (unpublished) | 0/207 (all tumours, 4 hospitals) | No cases | 0/52 |

* Figures for both sexes together.

† Estimated total: total carcinomas in men plus an estimate of the number of sarcomas in men from the total in both sexes.

variation in incidence. The drinking of home-distilled spirit is common in parts of Africa but there is no geographical evidence for an association with cancer of the oesophagus. The drinking of beer made from local cereals or fruits is widespread,

FIG. 1.—Proportional frequency of cancer of the oesophagus.

and the distribution of maize and in particular beer made from maize is in many ways similar to the distribution of cancer of the oesophagus. Maize is an introduced crop in Africa and its spread as an ingredient of beer seems to coincide with the rise in the frequency of cancer of the oesophagus.

### GEOGRAPHICAL DISTRIBUTION

Fig. 1 and the accompanying table which serves as a key (Table I) summarize the evidence so far available for the distribution of cancer of the oesophagus in Africa. The figures on the map show the proportion of oesophageal tumours in men* relative to some estimate of the total number of malignant tumours. Where more than one estimate of frequency exists for a single centre all the series are listed in the table, but only the most recent is plotted on the map. The material presented is of a variable standard; several of the tumour counts are known to be incomplete in that they exclude the many cases diagnosed without histological confirmation, and the estimates of frequency from different areas are sometimes for periods more than a decade apart. However, there is no evidence that the pattern of distrbution could itself be an artefact of an inadequate reporting of cases, and the geographical variation shown on the map is so striking that the underlying differences in incidence must be large enough to override the limitations of the data.

The outstanding features of the geographical distribution are:

(1) The occurrence of areas of very high frequency in southern, central and East Africa;

(2) A very low frequency throughout West Africa; and

(3) Sharp gradients in frequency from very high to very low within southern, central and East Africa.

The evidence for each aspect of the distribution is examined in greater detail below.

### *Areas of high frequency in southern, central and East Africa*

In Cape Province, Johannesburg, the Transkei, Durban, Bulawayo, southern Malawi, and western and central Kenya oesophageal tumours are either the most common or the second most common type of cancer diagnosed in men, representing from 16% to almost 30% of all tumours (Table I; 47, 37, 45, 46, 43, 33, 30, 16, 23, 24).

The exact proportion and rank at each centre is partly determined by the frequency of other commonly occurring types of cancer which themselves show geographical variation. If the effect of these is eliminated by removing the more variable tumours from the totals, figures are obtained which allow more accurate comparisons of the frequency of cancer of the oesophagus between different areas (Cook and Burkitt, 1971). For example, in some towns in southern Africa lung tumours are now common, but they are still rare in rural southern Africa and throughout East Africa. In Malawi bladder cancer is almost as common as cancer of the oesophagus and Burkitt's lymphoma is fairly frequent. In western Kenya cancer of the penis and Burkitt's lymphoma are both relatively common. The effect of removing tumours of these sites from the totals is to increase the proportional frequency of cancer of the oesophagus in Johannesburg, Durban, Bulawayo, southern Malawi and Central Nyanza by from 3 to 8%†. The figure for central Kenya is relatively unchanged increasing from 16 to only 17%. Liver cancer which like cancer of the oesophagus is the commonest or second most common

* Except for the two figures for the Transkei which are for both sexes.

† Adjusted percentages: Johannesburg 33%; Durban 27%; Bulawayo 20%; southern Malawi 27%; Central Nyanza 28%.

type of cancer in many parts of Africa has not been omitted from the totals because it seems to be widespread throughout Africa south of the Sahara and to show relatively little geographical variation (Cook and Burkitt, 1971). For this reason the frequency of oesophageal tumours has been shown relative to the frequency of liver cancer at each centre (Table I; last column).

No figures are (as yet) available from the Transkei from which it is possible to estimate the proportional frequency of cancer of the oesophagus in men. However the ratio of male to female incidence is reported to be only *c.* 1·5 to 1 so that the figure of 18% at Frere Hospital which represents the frequency in both sexes together is probably only a slight underestimate (Table I; 46).

A very high frequency of 53% (frequency in both sexes) has been reported from the smaller, mission hospital at Glen Grey in the Transkei (Table I; 45) but it is difficult to interpret such an extreme figure without further information about the completeness of the records, or the admissions policy of the hospital, or the place of residence of each patient. The cases were found by a retrospective search through 8 years hospital records.

The situation is not entirely comparable, because Glen Grey is a small, isolated mission hospital, but the type of bias which can occur in hospital records is well illustrated from a detailed investigation which has been made of the high frequency area around Kisumu Provincial Hospital in western Kenya. An apparent proportion of 30% (frequency in both sexes) (Ahmed, 1966) was reduced to 24% when it was found that, because of the known interest of the Provincial Surgeon, patients with cancer of the oesophagus seemed to be referred to the Provincial Hospital from distant districts more frequently than patients with tumours of other sites (Ahmed and Cook, 1969). The principal catchment area of Kisumu Hospital is the Central Nyanza District and the figures from Kisumu are therefore presented only for those patients who were resident in this district (Table I; 15 and 16). The first entry for Central Nyanza is from the tumour registry kept personally by the former Provincial Surgeon (Ahmed, 1967, personal communication). The tumour registry kept by the subsequent Provincial Surgeon (D' Cunha, 1969, personal communication) has been supplemented by a search through all hospital records to ensure that tumours diagnosed in the medical wards are also included. In this most recent series the proportion of oesophageal tumours in men and women together is only 16%, a reduction of the original estimate by almost one half.

The oesophagus is still overwhelmingly the most common site of tumour growth in men in Central Nyanza. Twenty-five per cent of all male cancer patients had oesophageal tumours and there were almost three times as many patients with cancer of the oesophagus as with either liver or stomach tumours. However, it is possible that, even within the immediate catchment area around Kisumu, oesophageal cancer patients are disproportionately attracted or admitted to the hospital. At the two neighbouring hospitals of Maseno and Kaimosi the proportion of oesophageal tumours was slightly lower than at Kisumu (only 22%) and the ratio of oesophageal to liver tumours was much less (only 1·3 : 1). The evidence for the true proportion is inconclusive. Maseno and Kaimosi are much smaller hospitals and the figures are less likely to be biased by differential referral, but they also have less good diagnostic facilities so that fewer of the diagnoses of any types of cancer had histological confirmation.

The high frequency area around Nairobi seems to extend throughout central Kenya and to affect the Meru and Embu tribes as well as the Kikuyu and Kamba

(Cook and Burkitt, 1970, unpublished report). Further evidence presented in Table I shows that the ratio of oesophagus to liver tumours was the same in the series of tumours reported from all the up-country hospitals in the central and north-eastern Districts as among the Kikuyu and Kamba patients diagnosed at the Kenyatta National Hospital in Nairobi (Table I; 23 and 24). The frequency in central Kenya is probably slightly less than the very high frequency in western Kenya. The straighforward percentage frequency is less (only 16% compared with 20–25%) and has been shown above to alter little when other geographically variable tumours are omitted from the series. In addition cancer of the oesophagus is no more common than cancer of the liver whereas in western Kenya it is reported to be up to three times as common.

The only other high frequency shown on Fig. 1 is at Port Sudan on the Red Sea (Table I; 2) but at this hospital the majority of oesophageal tumours diagnosed in both men and women seem to be in the cervical oesophagus (Nabri, 1966–69, personal communication) whereas in East and southern Africa it is the middle and lower thirds of the oesophagus which are most commonly affected (Ahmed, 1966; Burkitt and Cook, unpublished data; Higginson and Oettlé, 1960; Schonland and Bradshaw, 1968).

*Low frequency area in West Africa*

Only 4 cases of cancer of the oesophagus occur among almost 4000 tumours reported in the published series from West Africa. However the data from West Africa are both less complete and less recent than most of the material from the east and south of the continent. The figures from Senegal, Cameroon and the Congo include only tumours which have been diagnosed by histology. Generally in Africa estimates of the frequency of cancer of the oesophagus based on histological series are very misleading because the medical facilities of most hospitals are so poor that doctors tend to diagnose oesophageal tumours on clinical grounds without ever seeking histological confirmation (Burkitt, Hutt and Slavin, 1968; Cook and Burkitt, 1970, unpublished report). However, although the biopsy rate for cancer of the oesophagus is as low as 20% in the up-country hospitals of East Africa, in the capital cities of Africa, which each tend to have one hospital that is the showpiece of the country and where the facilities compare well with those of any hospital in the western world, the level is much higher (50% in Johannesburg; 75% in Kampala (Higginson and Oettlé, 1960; Kampala Cancer Registry, unpublished data, 1963–66)). Even if cancer of the oesophagus at the up-country hospitals of Senegal, Cameroon and the Congo was totally unrepresented in the histological series because a biopsy specimen had not been taken from suspected cases, any case diagnosed in the towns of Dakar, Douala, Brazzaville and Stanleyville (now Kisangani) would be far more likely to have had histological confirmation (Table I; 64, 68, 70 and 71).

In Ghana the evidence of the histology series is supported by the autopsy records (Table I; 65). Biopsies may be rare, but there is far less reason why patients with cancer of the oesophagus should selectively escape post-mortem examination. However, only one case was found out of a total of 149 malignant tumours.

The three series from West Africa which include cases diagnosed by all methods (from Ibadan, Ilesha and Lambarene) tell the same story—that cancer of the oesophagus is usually totally absent and never accounts for more than 1% of all malignancies (Table 1; 66, 67 and 69).

In all the series from West Africa other internal tumours are well represented. Stomach cancer has a proportional frequency of 9% in men in Ibadan and 16% in Ilesha and liver cancer is everywhere common, again helping to confirm that the low frequency of cancer of the oesophagus is genuine.

*Gradients of frequency in southern, central and East Africa*

Areal gradients in the frequency of cancer of the oesophagus undoubtedly exist in southern Africa, but the range of frequency is difficult to establish with accuracy because records from different centres are seldom published for the same period and because there is strong evidence from South Africa that there has been a temporal increase in frequency over the past few decades. Where possible, comparisons are made only from data for similar years.

A study of cancer among all African residents of Johannesburg from 1953–55 showed cancer of the oesophagus to have a proportional frequency in men of 11% (Higginson and Oettlé, 1960). The records of 7 hospitals serving rural areas of the Northern Transvaal were examined in the course of the same survey and showed a frequency of only 2% (Table I; 39). Liver and lung tumours on the other hand were diagnosed with a frequency similar to that in Johannesburg (liver 22% compared with 23% and lung 7% compared with 8%).

More recent figures from South Africa show that the gradient still exists. The figure of 28% for Johannesburg in 1962–64 can be compared with a frequency of 6% observed at the hospital of Acornhoek in the Northern Transvaal between 1957 and 1966 (Table I; 40).

Oettlé followed the original study of frequency with a postal questionnaire to all general hospitals in southern Africa to see whether other similar gradients could be established (Oettlé, 1963). He asked for the number of oesophageal tumours to be expressed relative to the number of hospital beds and found a ratio which varied from 25·8 per 100 beds in the Transkei and 25·4 in Tembuland (immediately to the north of the Transkei) to only 0·2 in Swaziland. Since no information is available about the total number of malignancies diagnosed at each hospital it is not possible to make direct comparison between Oettlé's figures and any quoted so far in the present paper. It is also difficult to know just how much reliance can be placed on the regional comparisons made within the paper. Oettlé himself drew attention to some of the deficiencies of the figures—the fact that no attempt was made to eliminate duplicates occuring in the records from the readmission of a patient to the same hospital or from multiple attendances at several hospitals, and the fact that in some towns the majority of cases had been referred from other parts of South Africa. (In Cape Town for example about 34 cases were seen each year, but the majority of those patients had been referred from the Transkei *c.* 700 miles away.) However, a more serious source of bias and one which is not mentioned, is that the denominator (the number of hospital beds) itself shows considerable variation relative to the population at risk. In the Transkei there were only 0·35 hospital beds per 100 population whereas in Johannesburg there were 4·5 and in Cape Town 9·3. The number of hospital beds is specified in the paper only for those hospitals which participated in the survey. In Johannesburg, the Transkei and Cape Town all hospitals participated. In Durban only 2 out of the 4 hospitals co-operated and these 2 alone gave a ratio of 11·6 beds per 100 population. In defending the choice of this denominator rather than the alternative sometimes used (the number of hospital admissions) it is stated that, however variable the number of admissions from other causes, all cases of cancer of the oesophagus are likely to be admitted " if only for diagnosis or special feeding before being referred to another centre ". If then there are only one-thirtieth the number of beds relative to the total population (the difference between the Transkei and Cape Town or Durban) the number of oesophageal tumours will be heavily concentrated relative to the number of beds. Considerable doubt therefore falls on the very high ratios found in the Transkei and Tembuland. There is no disputing the fact that the incidence there is very high, but how high relative to other parts of South Africa, which it was the aim of the study to establish is still not clear. The only mention made of this possible error is that the large number of beds could account for the surprisingly low ratio of cases to beds in Durban where the disease was stated by doctors to be common. In view of the relative frequencies now established for Johannesburg and Durban of 28% and 20% (Table I; 37 and 43) the sixfold difference indicated by the ratio of cases to beds (Johannesburg 6·5 per 100 beds and Durban 1·0) is definitely misleading.

Although the figures given for the towns and for the apparent high frequency rural areas are difficult to interpret, the rural areas which show a very low ratio in Oettlé's study (the western and south-eastern Cape, Emboland, Natal, Zululand, the Transvaal, the Orange Free State, Leshoto and Bechuanaland) were probably genuine areas of low frequency and have been marked as such on the map (Table I; 48–57).

Just across the border from the apparent low frequency area of Zululand, the proportional frequency in men at Lourenco Marques in Mozambique from 1956–60 was only 2% (Table I; 42). The liver cancer frequency at Lourenco Marques was exceptional (66% of all malignancies in men), but if this figure is reduced to a level more usual for Africa (say 25%) and the other geographically variable tumours (cancer of the lung, penis and bladder) are omitted from the series, the proportion of oesophageal tumours is only increased to 5% which is still far below the high frequencies of the Transkei and Johannesburg.

Burrell (1962, 1969) and Rose (1967) reported a heavy concentration of cases in some areas within the Transkei and wide tracts of territory which had a very much lower incidence. However, no figures have as yet been presented from which it would be possible to assess the evidence for this local variation. Further investigation of the situation in the Transkei (Marais and Drewes, 1962) was reported to indicate a clear clustering of areas of high incidence along a broad belt of sedimentary rocks of one geological period (the Beaufort series) and a much lower frequency both on earlier and later sedimentary rocks and on igneous out-crops. Again the total pattern of distribution of cancer of the oesophagus was not presented; instead isolated localities with a very high frequency were picked out on the map.

Within East and Central Africa the pattern of changing frequency is more fully documented. In particular there is a marked contrast between western Kenya and Uganda. When this gradient was first analysed in detail (Ahmed and Cook, 1969) cancer of the oesophagus represented only 2% of all malignancies in the most recent series from the area around Kampala in Uganda (Table I; 4) and estimates of incidence indicated that the frequency was between 9 and 23 times higher in the area around Kisumu less than 200 miles to the south-east.

Since 1964 the majority of hospitals in East Africa have sent monthly reports of all cases of 7 selected types of cancer (Cook and Burkitt, 1970, unpublished report). A map of the distribution of cancer of the oesophagus by hospital suggests strongly that the change in frequency occurs at the national boundary between Kenya and Uganda. All the hospitals in western Kenya showed a high frequency, while in eastern Uganda there were very few cases diagnosed at any hospital (Table I; 10).

The figures for north-east and south-west Uganda shown on Fig. 1 are also taken from the " 7 tumours " study. In the north-east there was no case of cancer of the oesophagus reported (Table I; 9) and in the south-west the one case diagnosed represented only 0·5% of the 7 tumours (Table I; 11). The proportion relative to all cancer would be negligible if the full series of malignancies was available. No cases were reported from the adjacent territories of Rwanda and Burundi and the eastern Congo Republic (Table I; 12 and 72).

The proportion around Kampala now seems to be over 5% (Table I; 6). At Masaka, less than 75 miles to the west the frequency during the same period (1967–68) was only 1% (Table I; 7).

A gradient of frequency also occurs between western Kenya and the area of Tanzania immediately to the south of the border. However, there are few hospitals in this area and there is less reliable information than for the gradient between western Kenya and Uganda. At Shirati in Tanzania the frequency is definitely very low (Table I; 14) but it is not clear whether the change occurs abruptly at the border or whether there is a gradual transition through South Nyanza District to the high frequency area of Central Nyanza. The doctor at the small hospital of Kilgoris in Kenya which is the nearest hospital to Shirati across the border sees 6–10 oesophageal tumours a year (Tellegen, 1970, personal communication) suggesting an abrupt transition, but the crude incidence among patients from South Nyanza at Kisumu Provincial Hospital was very low although the ratio of oesophageal to other tumours was still high (Ahmed and Cook, 1969), and this could indicate either a more gradual or an abrupt change. Without more detailed knowledge of the catchment area of the Provincial Hospital and of the pattern of referral of cancer patients from South Nyanza the exact nature of the gradient remains unclear.

Between the high frequency areas of western and central Kenya lies the eastern branch of the great Rift Valley. The combined figures from the widely dispersed hospitals suggest that the incidence there is less than in the neighbouring areas to the east and west (Table I; 21).

Within Tanzania there are two areas of moderate frequency where cancer of the oesophagus represents 9 or 10% of all malignancies in men. The West Lake area (Table I; 13) grades rapidly westward to the low frequency area of Rwanda and Burundi and northward to the low frequency around Masaka. From the Tanga Province area on the coast of northern Tanzania (Table I; 25) the frequency declines inland to total absence around Mvumi in the centre of the country (Table I; 26).

The " seven tumours " study showed a very low frequency in the Mtwara and Mbeya Provinces of southern Tanzania (Table I; 27 and 28). (Where cancer of the oesophagus represents between 0·5% and 2·0% of the 7 types of cancer, which are the only available denominator, the frequency on the map has been marked " very low " (VL) rather than as a specific percentage of all tumours. The actual proportion in both provinces is probably around 1%.).

The low frequency in Mbeya Province at the head of Lake Nyasa grades southwards to the very high frequency area of southern Malawi at the foot of the lake where cancer of the oesophagus represents 20% of all malignancies in men, is the most common tumour in men and is 1·4 times as common as liver cancer (Table I; 30). In northern Malawi (Table I; 29) the proportion is only 8%, liver cancer is over twice as common and 5 other types of cancer were diagnosed more frequently. The distance between southern Malawi and Mbeya Province is about 400 miles so that the gradient, though similar in extent is less sharp than that between Kenya and Uganda.

A preliminary study of cancer frequency in Zambia, made by touring all hospitals and asking doctors to estimate how frequently they saw tumours of different sites, and by examining the place of residence of cancer patients attending Lusaka Central Hospital (McGlashan, 1969), indicated that the pattern in Malawi is repeated in adjacent eastern Zambia. The frequency was reported to be very high at all hospitals in the south-east of the country while in north-eastern Zambia doctors at 11 hospitals scattered over 6000 square miles reported that they never saw a case of cancer of the oesophagus.

### CHANGING FREQUENCY IN TIME

### *Increases in frequency in southern Africa*

In much of southern Africa the high frequency of cancer of the oesophagus seems to be a recent phenomenon. The number of oesophageal tumours in men diagnosed at the Baragwanath hospital in Johannesburg showed a sevenfold increase between the early 1950s and early 60s (Table I; 36 and 37). If allowance is made for the 2·5-fold increase which occurred in the total number of malignancies this represents an actual 5·3-fold increase. It seems that the increase is genuine and not merely an artefact of a possibly ageing population since other sites which show a comparable or greater rise of frequency with age, stomach, colon and prostate for example, have not shown a comparable increase in time. Cancer of the lung has shown a twofold increase, but this could have been expected from the widespread increases which have taken place elsewhere in the world. A much earlier series, provided by the hospital which preceded the Baragwanath in Johannesburg (Table I; 34), indicated that around 1930 cancer of the oesophagus was practically unknown, representing at that period only 2% of all malignancies. Stomach cancer was then 6 times and liver cancer 12 times as common as oesophageal cancer. By the 1950s, if allowance is made for the very large difference in the total number of tumours diagnosed at the two hospitals, cancer of the oesophagus had shown a 4·8-fold increase, and was second in frequency only to liver cancer. By the early 1960s it was the most common type of cancer in men.

Burrell reported that cancer of the oesophagus was probably unknown in the Transkei 25 years before his survey (Burrell, 1962). He gained this impression from discussion with local doctors who had worked for many years in the area and by asking elderly residents to recall their previous knowledge of the disease. In an area where cancer of the oesophagus is now so common that the local people have their own name for it (Xhosa, *umhtaza wombiza,* chronic ulceration of the gullet), and "illiterate laymen confidently diagnose the condition" (Oettlé, 1964), this is probably a reliable indication of change.

At the Frere hospital, East London, oesophageal cancer had reportedly only been recognized in recent years (Burrell, 1957). No figures are available from which it is possible to assess the extent of the change but, in the neighbouring Ciskei, cancer of the oesophagus represented less than 1% of all malignancies diagnosed between 1913 and 1933 at Lovedale mission hospital (Table I; 44). The more recent records of the Lovedale hospital are not published, but it is reported that the disease is now common (Rose, 1967). The number of patients, however, is said to be smaller than expected because long waiting lists result in the selection of patients on the basis of " treatability ". (This conflicts with the assumption made by Oettlé and quoted above that all suspected oesophageal cases would be admitted to South African hospitals at least for confirmation of diagnosis.).

In Durban there has been a five to sixfold increase in the number of cases dignosed in men between the early 1950s and mid-1960s (Schonland and Bradshaw, 1968). There is no indication of the extent to which all malignancies have increased in frequency.

At Edenvale Hospital, Pietermaritzburg in Natal there was a sixfold increase in the number of hospital admissions for cancer of the oesophagus between 1953 and 1964 (Coetzee, 1966). Again there is no indication of any change in the number of admissions for all malignancies and no figures from which the present relative frequency can be estimated.

At Bulawayo cancer of the oesophagus appears to have increased relative to all tumours (Skinner, 1967).

In the areas of South Africa where the present frequency is less dramatically high there is nevertheless some evidence of an increase in recent years. Swaziland before 1962 reported the lowest ratio of cases to beds in Oettlé's (1963) study. The proportion of beds to population was moderate by South African standards and so could not have been responsible for an unduly low ratio, but by 1964–68 cancer of the oesophagus represented 10% of all malignancies in men and was second in frequency only to liver cancer (Table I; 41).

In the rural Northern Transvaal the increase may just be beginning. At Elim hospital between 1926 and 1935 there was one oesophageal tumour out of a total of 84 malignancies (Des Ligneris, 1936). In 1953–55 Elim was one of the 7 rural hospitals which together still reported a frequency of only 2% (Table I; 39). At Acornhoek hospital between 1957 and 1966 the frequency was 6% (Table I; 40). Statistically this proportion is not significantly different from the percentage reported by the 7 hospitals and would only provide firm evidence of an increase if all 5 cases had occurred in the last few years of the study. However the indication of an upward trend from Acornhoek is strengthened by reports of a recent increase at 2 of the rural hospitals (Pretoria and Pietersburg) which showed a low frequency in the 1953–55 study (Oettlé, 1964).

*Increases in frequency in East Africa*

In East Africa there is very little data from which it is possible to estimate cancer frequency before the 1960s, but the scattered fragments do give some indication of increases in frequency.

Up to the mid-1930s the records of the central pathology laboratory in Nairobi which provides a histology service for the whole of Kenya contained only 2 oesophageal tumours, a frequency of 0·04% (Table I; 22). This series must be regarded with all the caution urged above for the interpretation of the histological series from West Africa, with the added possibility that 40 years ago biopsies may have been taken from oesophageal tumours even less frequently than today. However, other internal tumours were represented in the series in greater number (5 stomach tumours and 42 cases of cancer of the liver) and the figures provide strong indication of a former very low frequency.

At Maseno hospital in western Kenya, 13 miles north-west of Kisumu, a retrospective search has been made through case sheets dating back to 1926 (Table I; 17, 18 and 19). If the periods before and after 1950 are compared there has been a 1·6-fold increase in the frequency of cancer of the oesophagus after allowance for the increase in all malignancies. However, before 1950 the frequency seems to have been fairly constant (at *c*. 14% of all tumours) since the beginning of recording. The numbers are very small in the earliest series and the recording of other types of cancer which are less easy to recognize on clinical grounds could have been less efficient in the early days. An increase may have occurred but the extent is unknown.

As in the low frequency area of the Northern Transvaal there is some indication of an increase around Kampala in Uganda. Before 1960 the proportion of oesophageal tumours was only 2·5% in male residents of Kyadondo County; 11 other types of cancer occurred more frequently and the ratio of oesophageal to liver tumours was only 0·15 : 1 (Table I; 4). Only genuine residents were included in the study and addresses were checked by follow-up visits to the home of almost all patients who gave the county as their home-address. Such a rigorous check has not been made in more recent years and the later figures from the Kampala Cancer Registry are not therefore completely comparable with the earlier series. The figures presented in Table I are given for what seems to be the local catchment area of Mulago Hospital, Kampala (East and West Mengo Districts); an area (including Kyadondo County) which has been defined by plotting the crude incidence of all types of cancer diagnosed at Mulago from all over Uganda. Calculations have also been restricted to the local tribe, the Buganda, because with members of other tribes there is no way of telling whether they have moved permanently to the area, or have merely come to seek treatment and have given the address of friends or relatives near Kampala. The proportion of oesophageal tumours in 1963–66 was 4·1% and in 1967–68 5·3% by which time cancer of the oesophagus had moved up from twelfth place to being the seventh most common type of cancer in men. No other type of cancer has shown a comparable rise in frequency.

There are no published reports from West Africa of any increase in frequency, but there is an indication that cancer of the oesophagus may now be appearing in the west of the continent from the verbal report of the surgeon at Kinshasa (Jain, 1970, personal communication) who in the past 3 or 4 years has seen a total of perhaps 8 cases compared with virtually none in previous years.

In view of the apparent recent occurrence of cancer of the oesophagus in Africa and the lack of any evidence that it was common anywhere in the continent 30 or 40 years ago, it is of interest to note the report made as long ago as 1924 by a missionary doctor from the high incidence area of north China that " cancer of the breast is very common, also cancer of the cervix. Next comes cancer of the oesophagus " (Davies, 1924). The high standard of observation in many mission hospitals makes it unlikely that a common cancer throughout much of Africa could have been completely overlooked in the past.

*Estimations of incidence relative to the population at risk*

All the figures discussed so far have been proportional frequencies because this is all that is available from most of Africa (Cook and Burkitt, 1970, unpublished report). However, reliable incidence figures have been published for a few centres and these indicate both that the frequency in parts of East and South Africa is among the highest known anywhere in the world and that the range of frequency within Africa is almost hundredfold compared with say liver cancer for which the range is only about fivefold (Cook and Burkitt, 1971). All estimates of incidence given below are age standardized for the limited age group 35–64 (Doll and Cook, 1967).

In 1953–55 the incidence in men in Johannesburg was 21·8 per 100,000 (Higginson and Oettlé, 1960; Doll, 1969). The more recent figures from Baragwanath hospital (Robertson, 1969) cannot be used directly to make comparable estimates of incidence because the earlier series included only cases diagnosed among residents of Johannesburg and was based on records obtained from many different sources, whereas the Baragwanath series includes only hospital inpatients and there has been no attempt to distinguish long-term residents of Johannesburg from those who have come temporarily to the town to seek work, possibly from as far afield as Malawi or Mozambique. However, if it is assumed that the increase in frequency demonstrated by the hospital series (Table I; 36 and 37) has affected residents and non-residents equally, and also that it has occurred equally at all ages, the incidence of 21·8 per 100,000 found in 1953–55 would have increased to 136 per 100,000 by 1963–65.

From 31 cases of cancer of the oesophagus occurring in one locality of East London between 1952 and 1956 Burrell calculated age specific incidence rates (Burrell, 1957) which give an incidence in men of 100·8 per 100,000. Rose gives age specific incidence rates for the Butterworth District of the Transkei (based on 151 cases seen in the 10 years from 1955 to 1964) (Rose, 1967) which (by interpolation since the actual rates are given for age groups which are not directly comparable) suggest an incidence in men of 246·2 per 100,000. This figure is only slightly lower than the highest reported incidence from any part of the world, in Gurjev in Kazakhstan (Doll, 1969).

From the Bulawayo figures the comparable incidence in 1963–65 was 125·8 (Doll, Muir and Waterhouse, 1970) and for Durban in 1964–66 98·9 (Doll, 1969). The rates for both towns are based on a careful scrutiny of all possible sources of cancer records (Skinner, 1967; Schonland and Bradshaw, 1968).

The observed incidence in men in the Central Nyanza District of West Kenya, based on hospital admissions at Kisumu Provincial Hospital in 1965 and 1966, was 38·7. Adjustment of the data to allow for possible under-reporting of all types of cancer suggested that the actual incidence could be as high as 169 per 100,000 (Ahmed and Cook, 1969).

The only estimates of incidence from areas of lower frequency are from Ibadan, Lourenco Marques and Kampala. At Ibadan in 1962–64 the incidence in men was 2·6 per 100,000 (Edington and Maclean, 1965; Doll, 1969) and at Lourenco Marques in 1956–60, 11·8 (Prates and Torres, 1965; Doll, 1969). The highest estimate of incidence from the Transkei is 95 times the incidence in Ibadan.

The figure of 5·5 per 100,000 from Kampala in 1954–60 (Davies *et al.*, 1965; Doll, 1969) would have increased to 11·8 by 1967–8 if adjustment is made for the increase in the proportional frequency described above.

### SEX RATIO

### *The frequency of cancer of the oesophagus in women*

The frequency of cancer of the oesophagus among women in Africa is everywhere less than in men, but there is considerable variation in the extent of the difference and evidence that in some areas the relative frequency is changing.

Table II shows the ratio of male to female incidence for those areas where it has been possible to make meaningful comparisons. The number of entries is far fewer than in Table I partly because the areas of lowest frequency have been omitted and partly because the frequency in the two sexes can only be compared where there is some information about the structure of the population at risk. A straightforward ratio of the number of cases in men and the number in women would be misleading because of the great variation in the number of men and women in the total population. A current feature of life in Africa is the mass migration of men to the new and rapidly developing towns to find work. This takes place on a semi-permanent basis with only occasional visits to the wife and children who have been left with responsibility for cultivating the family farm. The result is that the sex ratio (M/F × 100 for the age group 35–64 or the nearest approximation to this age group) is between 85 and 95 in most rural areas, and anything from 130 (East London 1952–56) to 612 (Nairobi, 1962) in the towns. The ratios for oesophageal cancer in Table II, therefore, have either been based on estimates of age standardized incidence or are ratios of the absolute number of cases adjusted for the sex ratio of the population at risk.

TABLE II.—*Ratio of Male to Female Incidence of Cancer of the Oesophagus* (*Age* 35–64)

| Locality | Source of data | Date | Ratio |
|---|---|---|---|
| Johannesburg | Higginson and Oettlé, 1960 | 1953–55 | 11·0 : 1 |
| Johannesburg | Robertson, 1969 | 1962–64 | 5·3 : 1 |
| East London | Burrell, 1957 | 1952–56 | 2·0 : 1 |
| Transkei (Butterworth and Willowvale hospitals) | Rose, 1967 | 1955–64 | 1·5 : 1 |
| Durban | Schonland and Bradshaw, 1968 | 1964–66 | 3·1 : 1 |
| Bulawayo | Skinner, 1967 | 1963–65 | 7·5 : 1 |
| West Kenya | Ahmed and Cook, 1969 | 1965–66 | 11·8 : 1 |
| Central Kenya (Kikuyu and Kamba tribes) | Kenya Cancer Registry, 1970 (unpublished data) | 1968–69 | 4·4 : 1* |
| Kyadondo County, Uganda | Davies *et al.*, 1965 | 1954–60 | 1·6 : 1 |
| West Lake Province, Tanzania | Cook & Burkitt, 1971 | 1964–69 | 2·3 : 1*† |
| Northern Malawi (N of 13° S) | Cook and Burkitt, 1971 | 1968–69 | 2·5 : 1*† |
| Souther Malawi (S of 13° S) | Cook and Burkitt, 1971 | 1968–69 | 10·8 : 1*† |

* Ratio of cases adjusted for the sex ratio of the population at risk.
† Ratio of incidence of cases at all ages.

The ratios vary from 11 or 12 : 1 in southern Malawi and west Kenya to only 1·5 : 1 in the Transkei. There is no association between the absolute frequency and the sex ratio. Around Kampala (Kyadondo County) both the incidence and the sex ratio are low. In Bulawayo and west Kenya both are high. In the Transkei a high frequency is accompanied by a low sex ratio.

### AETIOLOGICAL HYPOTHESES

Studies in many parts of the world have indicated that the consumption of alcoholic drinks can be a factor in the development of cancer of the oesophagus. This is true in areas of high frequency such as north-west France or southern Africa (Tuyns, 1970; Higginson and Oettlé, 1960; Burrell, 1962), in areas of moderate frequency such as Puerto Rico (Martinez, 1969) and in areas of low frequency such as the United States or Sweden (Wynder and Bross, 1961; Wynder *et al.*, 1957). The mere quantity of alcohol consumed is not a sufficient factor to explain the enormous geographical variation in frequency throughout the world (Doll, 1967), and there are, moreover, areas of moderate or high frequency such as India or Iran where alcohol seems to play no part in the development of oesophageal tumours (Wynder and Bross, 1961; Kmet and Mahboubi, 1972).

In Africa the geographical pattern of alcohol consumption shows a similar lack of association with the distribution of cancer of the oesophagus. There are areas of very high oesophageal cancer frequency such as the Transkei where the consumption of distilled spirits is less high than in the urban areas of South Africa (Burrell, 1962), and there are areas such as southern and north-western Uganda where the drinking of home-made spirits is common (Uganda Government, 1963) but where the frequency of cancer of the oesophagus is low or very low (Table I; 6 and 8). However, there are sufficient pointers to alcoholic drinks to suggest that they could be carriers of a carcinogenic agent, or that they could play an important co-carcinogenic role in some area of the world, especially in those regions where cancer of the oesophagus is more common in men than women.

Various sources of contamination have been suggested, in particular lead, zinc, copper, or polycyclic hydrocarbons from the old oil or asphalt drums used as containers during fermentation and distillation, or from the discarded exhaust pipes which have been used as part of the distilling apparatus (Oettlé, 1964; Reilly and McGlashan, 1969; Rose, 1968, and McGlashan, 1969). Attention has also been drawn to additives, such as metal polish, apparently included in some distilled liquors in South Africa to give extra flavour and strength (Burrell, 1957). Many of these however are features of the preparation of drinks in urban areas and cannot explain the very high frequencies of cancer of the oesophagus in rural areas such as the Transkei (Oettlé, 1967). A survey of the methods of preparation of alcoholic drinks in a range of areas of differing frequency in Kenya and Uganda showed marked regional variation in the use of containers made of clay or metal and in the use of copper piping, but the distribution patterns showed no association with the distribution of cancer of the oesophagus (Cook *et al.*, 1971). There was also no evidence for the use of exhaust pipes as part of the distilling apparatus or for the inclusion of exotic additives in the drinks consumed.

McGlashan found levels of zinc in distilled spirit from Zambia which were far above the recommended limit of safety for drinking water (McGlashan, 1969), but evidence from animal experiments suggests that a deficiency rather than an excess of zinc causes damage to oesophageal tissue which might predispose to malignant change (Morrison and Sarret, 1958; Follis *et al.*, 1941).

A more promising lead was the report from a high frequency area of Zambia that nitrosamine compounds seemed to occur in spirits distilled locally from sugar and maize husks (McGlashan *et al.*, 1968). Many nitrosamines have been shown to be highly carcinogenic, and several are specific to the oesophagus causing tumours at this site by whichever route they are administered (Magee and Barnes, 1967). However, the early methods used for detecting nitrosamines in alcoholic drinks based on a general screening by polarography have since been shown to be too unspecific to be a useful indicator (McGlashan *et al.*, 1970; Foreman and Palframan, personal communication) and subsequent analysis of spirit samples from areas of high and low frequency in East Africa by gas–liquid chromatography and mass spectroscopy showed no evidence for the presence of nitrosamines down to a level of 0·1 ppm (Cook *et al.*, 1971).

An association has been reported from the Transkei between the place of residence of oesophageal cancer patients and the occurrence of plants affected with a disease caused by molybdenum deficiency (Burrell *et al.*, 1966). This particular deficiency leads to an accumulation of nitrates in plants and these could combine with naturally occurring secondary amines to produce nitrosamines in foodstuffs. Plants and prepared foods from the Transkei are currently being analysed for their nitrosamine content (Rose, 1967). However, it is difficult to see how carcinogenic agents in a commonly consumed food could account for the differences in the frequency of cancer of the oesophagus in men and women in other parts of Africa.

Several studies from different parts of the world have reported an association with low social class or with poor nutritional status, either in the oesophageal patients themselves or on a geographical basis (Wynder and Bross, 1961; Martinez, 1969; Kmet and Mahboubi, 1972). The association between sideropenia, the Plummer–Vincent syndrome and cancer of the cervical oesophagus in women is well established (Wynder *et al.*, 1957) but the role of specific deficiencies in the development of tumours in the thoracic oesophagus is imperfectly understood and needs further investigation. Malnutrition is widespread in Africa and occurs in areas where cancer of the oesophagus is very rare as well as in areas of high frequency, but it could be that deficiency of some kind is a necessary background factor for the development of cancer of the oesophagus.

One suggested explanation for the rise in frequency in the Transkei is that during the famine period of the 1931–33 drought there was a greatly increased consumption of the brown and red-brown sorghums grown in southern Africa which have a high tannin content and which could be carcinogenic (Morton, 1970). However, as will be shown in greater detail below, sorghum is almost everywhere giving way to maize as a major food-crop in Africa, and it seems unlikely that a short-term change in dietary habits could be responsible for so widespread an increase as has occurred in the frequency of cancer of the oesophagus. A study of the age distribution of epithelial tumours such as cancer of the oesophagus suggest that they are probably caused by regular and prolonged exposure to some carcinogenic agent over a period of many years (Cook *et al.*, 1969).

Several studies have shown a slightly increased incidence of tobacco-smoking in oesophageal cancer patients (Clemmesen, 1965; Wynder and Bross, 1961; Schwartz *et al.*, 1957; U.S. Department of Health, 1964) but, as with alcohol, the global pattern of consumption shows no association with the distribution of cancer of the oesophagus throughout the world. Smoking habits in different parts of Africa have not been studied in detail but there are scattered pieces of information which indicate a similar lack of association. Long-stemmed pipes were commonly smoked by both men and women in the Transkei (where oesophageal cancer is common in both sexes) (Burrell, 1957) and in western Kenya (where oesophageal cancer is common only in men). Cigarettes are now everywhere replacing traditional methods of smoking or are spreading where it was not formerly the custom to smoke. Lung cancer is following in their wake and has already become common in some of the bigger towns although it is still rare in most rural areas (Skinner, 1967; Schonland and Bradshaw, 1968; Robertson, 1969; Cook and Burkitt, 1971).

FIG. 2.—Traditional staple food crops in Africa (after Murdoch, 1960).

The distribution of cancer of the oesophagus in Africa is in no way similar to the distribution of cancer of the lung.

Other aetiological factors commonly invoked are those which cause mechanical trauma; excessively hot food and liquids swallowed down an oesophagus partially anaesthetized by home-distilled spirit (Burrell, 1957); silica particles from the grinding stone used to prepare flour (Rose, 1968); fish bones stuck in the oesophagus (D'Cunha, 1969, personal communication), and excessive intake of heavily spiced food (Martinez, 1969). However, whereas many of these look promising locally, none of them can account for the geographical distribution of cancer of the oesophagus throughout Africa nor more particularly for the distinctive features of the sex ratio and the changing frequency in time.

*Possible role of alcoholic drinks made from maize*

McGlashan in Zambia and Malawi showed a geographical association between the occurrence of cancer of the oesophagus and the drinking of spirit made from maize husks and sugar. He also found a negative association with the drinking of beer made from millet (McGlashan, 1969). In the survey of the preparation and consumption of alcoholic drinks in Kenya and Uganda, the areas of high frequency of cancer of the oesophagus in west Kenya were found to be areas where maize beer is consumed, while the areas of low or very low frequency in Uganda were areas of millet, sorghum, banana or honey beer (Cook *et al.*, 1971). Furthermore the use of maize for beer-making was found to be a recent custom, maize having replaced the traditional sorghum and millet beers of west Kenya.

Following these two leads material has been accumulated about the distribution of maize in Africa and in particular about the use of maize in the preparation of alcoholic drinks.

Fig. 2 shows the areas in which maize was the traditional staple crop (Murdoch, 1960). Though small in scale the map is based on an exhaustive study of the anthropological and historical literature about hundreds of different African tribes (Murdock, 1959). (By " traditional " Murdock in fact means since the advent of European literature about Africa, for maize is not an indigenous crop in the continent but was introduced by the Portuguese in the seventeenth century (Cole, 1961)).

It can be seen that there is a broad coincidence between the areas where maize was " traditionally " staple and the areas—in eastern South Africa, in Rhodesia, in south-eastern Zambia and in southern Malawi and in central Kenya—where cancer of the oesophagus is common. In West Africa where cancer of the oesophagus is virtually unknown, the traditional staples were yams, cassava, bananas and rice in the wetter areas and millet and sorghum in the drier zones towards the Sahara and Kalahari deserts. The only other part of the continent where maize is shown as the main staple is central Angola and this is at present an unexplored blank on the cancer map.

In the high frequency area of western Kenya the staples are shown as millet and sorghum. These have now been replaced by maize which spread widely in the area from the 1930s (Wagner, 1956; Ominde, 1968). In Kenya as a whole maize is now planted on half the cultivated land (O'Connor, 1966). It everywhere represents over 30% of the total food crop acreage and in districts of western Kenya the proportion is as high as 50, 60 or even 90% (Agricultural Census of Kenya 1960/61 (1962)). By contrast, in Uganda it is planted on less than 5% of the cultivated land (O'Connor, 1966; Uganda Atlas, 1962) (Fig. 3). This is not a variation which reflects differences in soil and climate because parts of Uganda are just as suitable for the growth of maize. However, since the 1930s the Ugandan government has discouraged the crop (McMaster, 1962). Without this curb it would probably have spread much more widely in the country. It is easier to grow than most other grains, requiring less weeding and being more resistant to fungus and to attack by birds. It also tends to yield more highly even under poor conditions of soil and climate. However, it has certain disadvantages which led the Ugandan government to adopt its restrictive policy. In particular it makes heavy demands on soil nutrients and ground-water and as a result the plants have to be widely spaced and the soil exposed to erosion during the early stages of the growing period. It is also less nutritious than other grains, containing a nicotinic acid anti-metabolite which gives rise to a lowered nicotinic acid level in the body which may lead to pellagra (Chick, 1951).

FIG. 3.—Crops representing more than 30% of total food crop acreage—early 1960s.

In the wetter areas of heavier soils in the south of Uganda around Kampala, where maize would grow particularly well, strong competition from other crops has also helped to restrict the actual area cultivated. The banana is the traditional staple of the area and has high prestige as a " good " food, while cotton which was encouraged by the government as a cash crop has now spread widely. The rainfall regime permits double-cropping during the year, cotton during one season and some other crop after the short rains. Maize has a long growth period and cannot be fitted efficiently into this annual system of planting (McMaster, 1962). In the other areas of Uganda, sorghum, millet and cassava are still the staple food crops (Fig. 3).

The government of Kenya, perhaps improvidently, actively encouraged the spread of maize with a maize marketing board which offered controlled prices at a higher level than could be obtained elsewhere (O'Connor, 1966). Maize has become exceedingly popular as a food, even to the point where there is a social stigma against the consumption of millet (Murdock, 1959), and as a result the cultivation of maize has been pushed into areas which are not ideally suited to its growth. In these drier areas of poorer soils, soil erosion has become a serious problem (Ominde, 1968).

The difference in government attitude to the growing of maize in Uganda and Kenya has been stressed at some length because a difference in administrative approach of this kind could be responsible for the peculiar change in the frequency of cancer of the oesophagus which seems to occur at the national frontier despite the fact that the boundary is in no sense a natural ethnic or geographical divide.

In Tanzania there has been no official policy towards the growing of maize, neither encouragement or discouragement, and the crop is gradually spreading in most parts of the country (O'Connor, 1966). The spread has been much slower than in Kenya largely because of the longer distances and consequent isolation of the scattered population groups.

There are fewer and less recent agricultural statistics from Tanzania, but it seems that in the low frequency area of Mara just across the border from western Kenya (Table I; 14) the principal crop at least until very recently was finger millet (O'Connor, 1966).

In the border area of southern Nyanza in Kenya where the evidence for the frequency of cancer of the oesophagus is inconclusive but where there may be a gradual decline in frequency towards the Mara district of Tanzania maize has spread less extensively and more recently than in the very high frequency area of Central Nyanza (Ominde, 1968).

In the low frequency areas of Mtwara and southern Mbeya in Tanzania (Table I; 28 and 27) the staple crops are respectively sorghum and bananas. In the moderate frequency area of Tanga Province (Table I; 25), where there is commercial development of sisal estates, maize has spread more widely than in any other part of the country being grown mainly by the sisal labourers or their wives (O'Connor, 1966).

The other moderate frequency area of Tanzania, the West Lake Province (Table I; 13), is the only area in Africa discovered so far in which cancer of the oesophagus is relatively common and where maize is apparently not the staple crop. Bananas are the principal crop here as in the adjacent Buganda Province to the west and north of Lake Victoria in Uganda (O'Connor, 1966).

In Malawi maize is the most important crop and almost every Malawi family grows some maize, but on the poorer soils in the centre and north of the country (where the frequency of cancer of the oesophagus is only moderate (Table I; 29)) more millet is grown than in the high frequency area of southern Malawi (Table I; 30) (Pike and Rimmington, 1965).

In the remaining two areas of central and eastern Africa where cancer of the oesophagus is practically unknown, north-eastern Zambia and Rwanda and Burundi (Table I; 31 and 12), the staple food crops are millet (Murdock, 1960) and cassava and sweet potatoes (Inforcongo, 1960).

In South Africa maize began to spread widely as a foodcrop toward the beginning of the nineteenth century (Cole, 1961). In central Kenya the change to maize seems to have taken place between 1890 and 1910 (Morgan, 1967). In both areas the change was too early for the spread of maize as a food to be directly associated with the recent rise in the frequency of cancer of the oesophagus. However, as has been shown above, a widely consumed food could also not be responsible for the differences observed in the frequency in men and women.

The association with alcoholic drinks made from maize in both Zambia and Kenya and the comparatively recent change to maize beer in western Kenya suggests that a product of the fermentation of maize may be important in the development of cancer of the oesophagus.

In South Africa the traditional indigenous grain, sorghum (known there as kaffircorn) though no longer valued as a food was retained well into the present century as an ingredient of beer, and sorghum continued to be grown specially for beer-making (Year Book of the Union of South Africa, 1954–55 and 1958; Cole, 1961).

Beer is mentioned in various accounts of South African diet, but the specific ingredients are not clear from the literature partly because the term " corn " is sometimes used to mean maize and sometimes merely as a general term for the local grain, and partly because it is often the custom to use one type of flour as the main ingredient of beer and another type for the fermenting agent (Cook *et al.*, 1971). In west Kenya maize is used as the main flour and sprouted finger millet as the fermenting agent. In south-west Uganda a beer is made entirely from malted sorghum but the portion used for the main flour and the portion used for the fermenting agent are cooked in different ways. In South Africa sorghum is said to be the basis of malt for " Kaffir-beer " (Year Book of the Union of South Africa, 1954–55; Cole, 1961) but it is not clear whether all the grain is malted or whether some other grain is used as the main flour.

In parts of the Transkei a beer made from maize is definitely now consumed (Warwick, 1971, personal communication) and a fermented porridge made from maize is eaten (Rose, 1967). Maize is mentioned as an ingredient of beer in both Johannesburg and the Northern Transvaal in the 1950s (Higginson and Oettlé, 1960; Sutherland, 1968). However, much more information is needed from southern Africa about the extent to which maize has replaced sorghum as the main ingredient of beer and about the date at which the change occurred. This could be especially revealing from the Transkei where sharp gradients of frequency from very common to very rare are said to occur within short distances.

Of particular interest also are the areas of southern Mozambique, Swaziland and Bechuanaland. In southern Mozambique cancer of the oesophagus is relatively uncommon but maize is shown as the staple foodcrop by Murdoch (Fig. 2). However the agriculture is diverse and many other crops are grown which could also be used for beer—coconut palms, sugar, bananas, pineapples and even cashew nuts, which are specifically mentioned by one author as a source of distilled liquor peculiar to the Mozambique coast (Grove, 1967). Prates and Torres (1965) only mention in general terms " fermented cereals and fruits " without specifying particular ingredients.

In Bechuanaland and Swaziland where cancer of the oesophagus is still rare or has increased more recently than in other parts of southern Africa, sorghum is said to have remained longest as a staple foodcrop before being replaced by maize (Shapera and Goodwin, 1937; Year Book of the Union of South Africa, 1954–55).

In East Africa beer is usually made from the staple source of carbohydrate—bananas in the low frequency areas of southern Uganda and Rwanda and

Burundi, millet in the north of Uganda and maize in western Kenya where it has replaced the traditional millet and sorghum beers during the past 30 or 40 years (Cook *et al.*, 1971; Inforcongo, 1960; Wagner, 1956).

The high frequency area of central Kenya was not covered in the recent survey of the production of alcoholic drinks in East Africa. Honey and sugar beers are the traditional drinks of the area (Msafiri, 1970, personal communication). Maize beer is now produced (Kanure, 1970, personal communication) but more evidence is needed about the extent of its manufacture. It is said that under British rule district officers in the area attempted to prohibit the growth of sugar in order to reduce beer-drinking (Lee-Woolf, 1970, personal communication). This would have encouraged a change to a grain-based beer.

In the moderate frequency area of the Kenyan Rift Valley the scattered groups of population have lived mainly by livestock herding, and grain has not played an important part in their economy. The traditional food of the Masai warriors was largely milk and blood, although millet and maize were eaten by other members of the tribe (Forde, 1934). Beer was brewed from honey. In the recent survey of alcoholic drinks honey and maize beers seemed to be consumed in roughly equal quantity (Cook *et al.*, 1971).

In the area around Kampala where it seems that the frequency of cancer of the oesophagus is just beginning to increase (Table I; 4, 5 and 6) maize is starting to spread despite the pressures against its introduction (McMaster, 1962) and some maize beer was found in the recent survey although the main drink is still banana beer (Cook *et al.*, 1971).

In West Africa there is no evidence from recent geographical literature that maize is seriously challenging the traditional staple food crops (Harrison Church *et al.*, 1967; Grove, 1967). The universal drink in the wetter areas from as far back as the eighteenth century is a wine tapped from various species of palm (the oil palm, *Elaeis guineensis*; the ngwo palm; *Raphia vinifera*; and *Raphia hookeriana*) (Bosman, 1705; Nigeria Handbook, 1926; Basden, 1938; Enaharo, 1965; Afolabi Ojo, 1966; Dickson, 1969).

### DISCUSSION

Evidence has been presented for the apparent association between the occurrence of cancer of the oesophagus in Africa and the use of maize as an ingredient of beer. In many respects the hypothesis can account for the geographical distribution of cancer of the oesophagus and for the increase in frequency over the past 30 or 40 years. More evidence however is needed on the latter point especially from southern Africa and also on the frequency of beer drinking in the two sexes. Reports from areas of East Africa where cancer of the oesophagus is much more common in men than women indicate that alcoholic drinks are consumed more frequently by men (Cook *et al.*, 1971) but in order to fit the hypothesis it would be necessary for women in the Transkei to take almost as much fermented maize as men, and for women in Johannesburg to have increased their beer consumption over the past 30 years (or to have continued drinking the traditional sorghum beer longer than the men).

Attention has been confined to beer, rather than spirit made from maize, because the consumption of beer is far more widespread than the consumption of spirit especially in some rural areas where cancer of the oesophagus is common. Beer has generally been rather overlooked in the literature on oesophageal cancer

(unless it has been fortified with some exotic ingredients) presumably because beer drinking in Africa is so widespread and apparently lacking in regional variation. This is not true if the ingredients are considered rather than the quantity consumed.

There is as yet no evidence that the consumption of beer made from maize could be of importance in other areas of the world where cancer of the oesophagus is common. In Central Soviet Asia and Iran wheat and barley are the staple grains and maize is not an important crop. In the Honan province of China wheat and millet are grown.

In the United States where the frequency of cancer of the oesophagus is low in whites and moderate in Negroes (Doll, 1967) almost 50% of the grain used in brewing and 70% of the grain used for the manufacture of distilled spirit is corn (maize) (Inglett, 1970). However, most of the alcohol consumed in the United States is commercially produced and although there is some illegal distillation of corn spirit there is no evidence for the widespread home production of maize beer as is common in Africa.

Evidence from other parts of the world suggests that factors may be locally important in the development of cancer of the oesophagus which have no bearing in other regions of high frequency. In France the local alcoholic drinks from Normandy and Brittany seem to be associated with a higher frequency than the alcoholic drinks of the rest of France (Tuyns, 1970). In northern Iran there is a very high incidence in which alcoholic drinks apparently play no part at all (Kmet and Mahboubi, 1972).

There is thus much circumstantial evidence from Africa that the consumption of beer from maize could be a factor in the development of cancer of the oesophagus there. It should be possible to further substantiate or to refute the hypothesis with more detailed epidemiological evidence of the same type as already collected —more information is needed in particular from the south of the continent—and this would seem the obvious first step accompanied by chemical analysis of beer samples for carcinogens or animal experiments to produce tumours.

My thanks are due to all the doctors and pathologists working in Africa whose work has made this paper possible, to Miss Irene Allen for helping analyse the data, to Miss Sandra Osborn for typing the text and drawing the diagrams, and to Miss Ella Wright for checking the typescript.

### REFERENCES

ABOUL NASR, A. L.—(1967) *Natn. Cancer Inst. Monogr.*, No. 25, p. 1.
AFOLABI OJO, G. J.—(1966) ' Yoruba Culture '. London (University of London Press).
AGRICULTURAL CENSUS OF KENYA 1960/61—(1962) Kenya (Government Printer).
AHMED, N.—(1966) *E. Afr. med. J.*, **43**, 235.
AHMED, N. AND COOK, P.—(1969) *Br. J. Cancer*, **23**, 302.
BASDEN, G. T.—(1938) ' Niger Ibos '. London (Seely Service & Co.).
BERMAN, C.—(1935) *S. Afr. J. med. Sci.*, **1**, 12.
BOSMAN, W.—(1705) ' A new and accurate description of the coast of Guinea, 1700–1702 '. London.
BURKITT, D. P., HUTT, M. S. R. AND SLAVIN, G.—(1968) *Br. J. Cancer*, **22**, 1.
BURRELL, R. J. W.—(1957) *S. Afr. med. J.*, **31**, 401.—(1962) *J. natn. Cancer Inst.*, **28**, 495.—(1969) *J. natn. Cancer Inst.*, **43**, 877.

BURRELL, R. J. W., ROACH, W. A. AND SHADWELL, A.—(1966) *J. natn. Cancer Inst.*, **36**, 201.
CAMAIN, R.—(1954) *Bull. Soc. Path. exot.*, **47**, 614.
CAPPONI, M.—(1953) *Bull. Soc. Path. exot.*, **46**, 605.
CHICK, H.—(1951) *J. trop. Med. Hyg.*, **54**, 207.
CLEMMESEN, J.—(1965) *Acta path. microbiol. scand.*, 1 suppl. 174.
COETZEE, T.—(1966) *S. Afr. J. Surg.*, **4**, 107.
COLE, M.—(1961) ' South Africa '. London (Methuen).
COOK, P. J. AND BURKITT, D. P.—(1971) *Br. med. Bull.*, **27**, 14.
COOK, P. J., COLLIS, C. H., FOREMAN, J. K. AND PALFRAMAN, J. F.—(1971) ' Cancer of the Oesophagus and Alcoholic Drinks in East Africa '. Privately published monograph.
COOK, P., DOLL, R. AND FELLINGHAM, S.—(1969) *Int. J. Cancer*, **4**, 93.
DAVIES, J. N. P., KNOWELDEN, J. AND WILSON, B. A.—(1965) *J. natn. Cancer Inst.*, **35**, 789.
DAVIES, S.—(1924) *Br. med. J.*, i, 131.
DENUES, A. R. T. AND MUNZ, W.—(1967) *Int. J. Cancer*, **2**, 406.
DES LIGNERIS, M. J. A.—(1936) *S. Afr. med. J.*, **29**, 478.
DICKSON, K. B.—(1969) ' A Historical Geography of Ghana '. London (Cambridge University Press).
DOLL, R.—(1967) ' Prevention of Cancer; Pointers from Epidemiology '. London (Nuffield Provincial Hospitals Trust).—(1969) *Br. J. Cancer*, **23**, 1.
DOLL, R. AND COOK, P.—(1967) *Int. J. Cancer*, **2**, 269.
DOLL, R., MUIR, C. AND WATERHOUSE, J., Editors—(1970) ' Cancer Incidence in Five Continents '. Berlin (Springer-Verlag), Vol. II.
EDINGTON, G. M.—(1956) *Br. J. Cancer*, **10**, 595.
EDINGTON, G. M. AND MACLEAN, C. M. U.—(1965) *Br. J. Cancer*, **19**, 471.
ENAHORO, CHIEF A.—(1965) ' Fugitive Offender '. London (Cassell).
FOLLIS, R. H., DAY, H. G. AND MCGOLLUM, E. V.—(1941) *J. Nutr.*, **22**, 223.
FORDE, C. D.—(1934) ' Habitat, Economy and Society '. London (Methuen).
GROVE, A. T.—(1967) ' Africa South of the Sahara '. London (Oxford University Press).
HARRISON CHURCH, R. J., CLARKE, J., CLARKE, P. J. H. AND HENDERSON, H. J. R.—(1967) ' Africa and the Islands '. New York (Longmans).
HICKEY, B. B.—(1959) *Ann. R. Coll. Surg.*, **24**, 303.
HIGGINSON, J. AND OETTLÉ, A. G.—(1960) *J. natn. Cancer Inst.*, **24**, 589.
INFORCONGO—(1960) ' Ruanda-Urundi; Economy 1 '. Belgian Congo and Ruanda–Urundi Information and Public Relations Office.
INGLETT, G. E. Editor—(1970) ' Corn: Culture, Processing and Products '. Connecticut (Avi Publishing).
KMET, J. AND MAHBOUBI, E.—(1972) *Science, N.Y.*, in press.
MCGLASHAN, N. D.—(1969) *Gut*, **10**, 643.
MCGLASHAN, N. D., PATTERSON, R. L. S. AND WILLIAMS, A. A.—(1970) *Lancet*, ii, 1138.
MCGLASHAN, N. D., WALTERS, C. L. AND MCLEAN, A. E. M.—(1968) *Lancet*, ii, 1017.
MCMASTER, D. M.—(1962) ' A Subsistance Crop Geography of Uganda '. The World Land Use Survey. Occasional Papers 2. Geographical Publications Ltd.
MACVICAR, N.—(1925) *S. Afr. med. Rec.*, **23**, 315.
MAGEE, P. N. AND BARNES, J. M.—(1967) *Adv. Cancer Res.*, **10**, 163.
MARAIS, J. A. H. AND DREWES, E. F. R.—(1962) *Ann. geol. Survey S.A.*, **1**, 105.
MARTINEZ, I.—(1969) *J. natn. Cancer Inst.*, **42**, 1069.
MORGAN, T. W.—(1967) ' Nairobi; City and Region '. Nairobi (Oxford University Press).
MORRISON, A. B. AND SARRET, H. P.—(1958) *J. Nutr.*, **65**, 267.
MORTON, J.—(1970) *Econ. Bot.*, **24**, 217.

MULLIGAN, T. O.—(1970) *Br. J. Cancer*, **24**, 1.
MURDOCH, G. P.—(1959) ‘ Africa: its Peoples and Their Culture History ’. New York (McGraw-Hill).—(1960) *Geogrl Rev.*, **50**, 523.
NIGERIA HANDBOOK—(1926) Lagos (Government Printer).
O'CONNOR, A. M.—(1966) ‘ Economic Geography of East Africa ’. Bell.
OETTLÉ, A. G.—(1963) *S. Afr. med. J.*, **37**, 434.—(1964) *J. natn. Cancer Inst.*, **33**, 383.—(1967) from ‘ The Prevention of Cancer ’, edited by R. W. Raven and F. Roe. London (Butterworth).
OMINDE, S.—(1968) ‘ Land and Population Movements in Kenya ’. Heinemann.
PIKE, J. G. AND RIMMINGTON, G. T.—(1965) ‘ Malawi; a Geographical Study ’. London (Oxford University Press).
PRATES, M. D. AND TORRES, F. O.—(1965) *J. natn. Cancer Inst.*, **35**, 729.
REILLY, C. AND MCGLASHAN, N. D.—(1969) *S. Afr. J. med. Sci.*, **34**, 43.
ROBERTSON, M. A.—(1969) *S. Afr. med. J.*, **43**, 915.
ROSE, E. F.—(1967) *Natn. Cancer Inst. Monogr.*, No. 25, p. 83.—(1968) *S. Afr. med. J.*, **42**, 334.
SCHONLAND, M. AND BRADSHAW, E.—(1968) *Int. J. Cancer*, **3**, 304.
SCHWARTZ, D., DENOIX, P. F. AND ANGUERA, G.—(1957) *Bull. Ass. fr. Étude Cancer*, **44**, 336.
SHAPERA, I. C. AND GOODWIN, A. J. H.—(1937) from ‘ The Bantu-speaking Tribes of South Africa ’, ed. I. C. Schapera, Routledge.
SKINNER, M. E. G.—(1967) *Natn. Cancer Inst. Monogr.*, No. 25, p. 57.
SUTHERLAND, J. C.—(1968) *Cancer, N.Y.*, **22**, 372.
THIJS, A.—(1957) *Acta Unio Int. Con. Can.*, **13**, 971.
TUYNS, A.—(1970) *Int. J. Cancer*, **5**, 152.
TUYNS, A. J. AND RAVISSE, P.—(1970) *J. natn. Cancer Inst.*, **44**, 1121.
UGANDA ATLAS—(1962) Uganda (Department of Lands and Surveys).
UGANDA GOVERNMENT—(1963) Report of the Spiritous Liquor Commission. Uganda (Government Printer).
U.S. DEPARTMENT OF HEALTH, EDUCATION AND WELFARE—(1964) ‘ Smoking and Health ’. Public Health Service Publ. No. 1103. Washington.
VINT, F. W.—(1935) *Lancet*, ii, 628.
WAGNER, G.—(1956) ‘ The Bantu of the North Kavirondo ’, vol. 2. Oxford University Press.
WYNDER, E. L. AND BROSS, I. J.—(1961) *Cancer*, **14**, 389.
WYNDER, E. L., HULTBERG, S., JACOBSON, F. AND BROSS, I. J.—(1957) *Cancer*, **10**, 470.
YEAR BOOK OF THE UNION OF SOUTH AFRICA NO. 28—(1954–55) Cape Town.
YEAR BOOK OF THE UNION OF SOUTH AFRICA NO. 31—(1958) Cape Town.

### APPENDIX

*Further notes on beer-making gathered during a visit to Africa in July and August* 1971

Maize is widely used in the Transkei for beer. A mixture of sprouted sorghum and sprouted maize is usually used for the fermenting agent and coarsely ground, unsprouted maize meal for the starchy adjunct. Most people remembered a time when much more sorghum was grown than at present although there seems no widespread memory, even in areas of apparently lower frequency, of a time when beer was made from sorghum alone so that the change may have occurred earlier than in Kenya. The change in planting habits is said, throughout southern Africa, to have occurred because as sorghum ripens the children are needed all day in the fields to scare away birds. Now the children go to school and maize is grown instead of sorghum. Much of the sorghum used as the fermenting agent is now bought from the store.

In the rural northern Transvaal, where the frequency of cancer of the oesophagus was low until more recently than in the Transkei, sorghum seems to have remained longer as a principal ingredient of beer (Quin, 1959; Stayt, 1931).

" Bantu Beer " is a widely sold commercial product in South Africa (104 million gallons in 1964 compared with an estimated 200 million gallons still made at home (Novellie, 1966)). The commercial beer resembles very closely the home-made product and is quite different from European beer. It is a thick, pinkish, cloudy liquid containing a high proportion of grain particles. It is still actively fermenting when sold so that it has to be packaged in a container with a valve, and as a result it has a shelf-life of only a few days (DeWit, 1971, personal communication; Diaber, 1971, personal communication).

The accompanying table (Novellie, 1968) shows that since the early 1950s the predominant grain used for Bantu Beer has been changed from sorghum to maize, a move taken largely to improve the keeping qualities of the product.

Constituents of Bantu Beer

| | | 1953–54 (%) | 1964–65 (%) |
|---|---|---|---|
| Sorghum | Kaffircorn malt | 56·3 | 36·9 |
| | Kaffircorn meal | 26·9 | 5·8 |
| Maize | Corn meal | 8·7 | 1·2 |
| | Brewer's grits | 8·3 | 56·0 |

In the last few years a similar commercial beer, " Chibuku ", also based on maize, has been introduced into Tanzania and Uganda.

The fermented maize porridge, " amahewa ", widely eaten in southern Africa, is made by the addition of wheat flour, but this causes a lactic acid fermentation and not an alcoholic fermentation (De Wit, 1971, personal communication).

In Swaziland maize is said to have been used for beer at least since the 1930s (Keen, 1971, personal communication; Beemer, 1939) and its use there may have been too early to be associated with the rise in frequency of cancer of the oesophagus.

In Mozambique, where the frequency of cancer of the oesophagus is still low (Torres and Bernarda, 1970), some maize beer has been made since early in the century (Junod, 1927) although a whole variety of fruit beers were also consumed. Present day observers gave conflicting opinions as to the role of maize beer in Mozambique today. An anthropologist who has lived among 3 of the major tribal groups reported that palm wine, and beer or spirit made from cashew fruit, moroela fruit, or from pineapples or oranges are the preferred drinks, and that maize beer would be made as a last resort only if these others were not available (Webster, 1971, personal communication). Dr. Torres felt that it was one of the drinks more commonly consumed (Torres, 1971, personal communication).

In the moderate frequency area of the West Lake Province of Tanzania there is some indication that home-made maize beer is still unknown (Haarer, 1958; Rald, 1969; Majaliwa, 1971, personal communication).

### REFERENCES

BEEMER, H.—(1939) *Bantu Stud.*, **13**, 199.
HAARER, A. E.—(1958) ' Modern Coffee Production '. London (Leonard Hill).
JUNOD, H. A.—(1927) ' Life of a South African Tribe '. London (Macmillan).
NOVELLIE, L.—(1966) *International Brewer and Distiller*, **1**, 27.

NOVELLIE, L.—(1968) *Wallerstein Labs. Commun.*, **31**, 17.
QUIN, P. J.—(1959) Food and Feeding Habits of the Pedi '. London (Oxford University Press).
RALD, J.—(1969) University College, Dar es Salaam, Research Paper No. 5.
STAYT, H. A.—(1931) ' The Bavenda '. London (Oxford University Press).
TORRES, F. O. AND BERNARDA, R. A.—(1970) *Revta Cienc. med., Lourenco Marques*, **3**, 45.